# Genetic Analysis of the Transition from Wild to Domesticated Cotton (*Gossypium hirsutum* L.)

**DOI:** 10.1534/g3.119.400909

**Published:** 2019-12-16

**Authors:** Corrinne E. Grover, Mi-Jeong Yoo, Meng Lin, Matthew D. Murphy, David B. Harker, Robert L. Byers, Alexander E. Lipka, Guanjing Hu, Daojun Yuan, Justin L. Conover, Joshua A. Udall, Andrew H. Paterson, Michael A. Gore, Jonathan F. Wendel

**Affiliations:** * Department of Ecology, Evolution and Organismal Biology, Iowa State University, Ames, IA 50011,; † Plant Breeding and Genetics Section, School of Integrative Plant Science, Cornell University, Ithaca, NY 14853,; ‡ Department of Crop Sciences, University of Illinois, Urbana, IL 61801,; § Plant and Wildlife Science Department, Brigham Young University, Provo, UT 84602, and; ** Plant Genome Mapping Laboratory, University of Georgia, Athens, GA 30602

**Keywords:** QTL, domestication, *Gossypium hirsutum*, cotton

## Abstract

The evolution and domestication of cotton is of great interest from both economic and evolutionary standpoints. Although many genetic and genomic resources have been generated for cotton, the genetic underpinnings of the transition from wild to domesticated cotton remain poorly known. Here we generated an intraspecific QTL mapping population specifically targeting domesticated cotton phenotypes. We used 466 F_2_ individuals derived from an intraspecific cross between the wild *Gossypium hirsutum* var. *yucatanense* (TX2094) and the elite cultivar *G. hirsutum* cv. Acala Maxxa, in two environments, to identify 120 QTL associated with phenotypic changes under domestication. While the number of QTL recovered in each subpopulation was similar, only 22 QTL were considered coincident (*i.e.*, shared) between the two locations, eight of which shared peak markers. Although approximately half of QTL were located in the A-subgenome, many key fiber QTL were detected in the D-subgenome, which was derived from a species with unspinnable fiber. We found that many QTL are environment-specific, with few shared between the two environments, indicating that QTL associated with *G. hirsutum* domestication are genomically clustered but environmentally labile. Possible candidate genes were recovered and are discussed in the context of the phenotype. We conclude that the evolutionary forces that shape intraspecific divergence and domestication in cotton are complex, and that phenotypic transformations likely involved multiple interacting and environmentally responsive factors.

The cotton genus (*Gossypium*) represents the largest source of natural textile fiber worldwide. Although four species of cotton were independently domesticated, upland cotton (*G. hirsutum* L.) accounts for more than 90% of global cotton production. Native to the northern coast of the Yucatan peninsula in Mexico, *G. hirsutum* is now widely cultivated across the globe (Wendel and Albert 1992). Domestication of *G. hirsutum* occurred circa 5,000 years ago, producing many phenotypic changes common to plant domestication, including decreased plant stature, earlier flowering, and loss of seed dormancy. An additional primary target unique to cotton domestication was the single-celled epidermal trichomes (*i.e.*, fibers) that cover the cotton seed. Cotton fiber morphology varies greatly in length, color, strength, and density among the myriad accessions that span the wild-to-domesticate continuum. As a species, *G. hirsutum* is highly diverse, both morphologically and ecologically, and has a correspondingly long and complex taxonomic history (Fryxell 1968, 1976, 1979, 1992) that includes the modern, cryptic inclusion of at least two distinct species (Wendel and Grover 2015; Gallagher *et al.* 2017). Truly wild forms of *G. hirsutum* (race *yucatanense*) occur as scattered populations in coastal regions of the semiarid tropical and subtropical zones of the Caribbean, northern South America, and Mesoamerica (Coppens d’Eeckenbrugge and Lacape 2014). These are distinguished from domesticated and feral forms by their short, coarse, brown fibers, as well as their sprawling growth habit, photoperiod sensitivity, and seed dormancy requirements, among others (Figure 1). Results from molecular marker analyses, including allozymes (Wendel and Albert 1992), restriction fragment length polymorphisms (RFLPs) (Brubaker and Wendel 1994), simple sequence repeats (SSRs) (Liu and Wendel 2002; Zhang *et al.* 2011; Tyagi *et al.* 2014; Zhao *et al.* 2015; Kaur *et al.* 2017; McCarty *et al.* 2018), SNP arrays (Hinze *et al.* 2017; Cai *et al.* 2017; Ai *et al.* 2017), and next-generation sequencing (Reddy *et al.* 2017; Fang *et al.* 2017c; Ma *et al.* 2018) have quantified genetic diversity and aspects of population structure among wild, feral, and domesticated stocks of the species, as well as the allopolyploid origin of the species. Notably, the allopolyploid origin of *G. hirsutum* includes a diploid species with no spinnable fiber, *i.e.*, the paternal parent derived from the fiberless Mesoamerican “D-genome” clade. The maternal progenitor of the allopolyploid lineage is derived from the African “A-genome” whose two extant species have been independently domesticated for fiber production.

**Figure 1:**
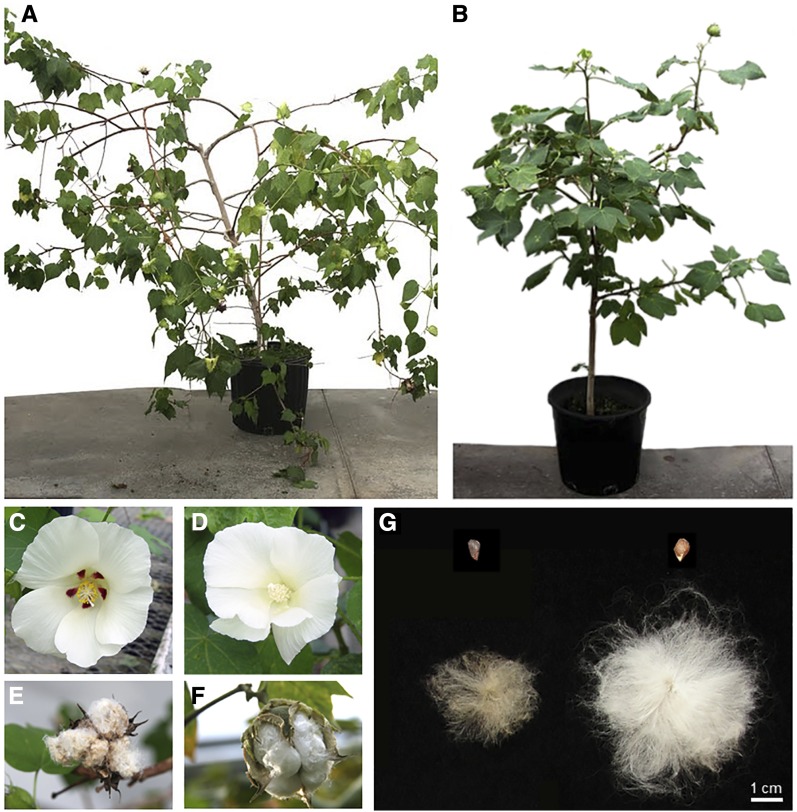
Morphological differentiation between *G. hirsutum* var. *yucatanense* TX2094 and *G. hirsutum* cv. Acala Maxxa. (A) Adult plant of TX2094, wild; (B) Adult plant of Acala Maxxa, domesticated; (C) TX2094 flower; (D) Acala Maxxa flower; (E) Open boll of TX2094; (F) Open boll of Acala Maxxa; (G) Ginned seed of TX2094 (top left) and Acala Maxxa (top right), and fiber of TX2094 (bottom left) and Acala Maxxa (bottom right). Photo credit: Kara Grupp & Mi-Jeong Yoo.

Recent advances have improved our understanding of the genetic changes targeted by humans during the several millennia of cotton domestication and improvement by evaluating gene expression differences that distinguish wild and domesticated cotton fiber, either globally or for a few key genes among accessions (Haigler *et al.* 2009; Bao *et al.* 2011; Kim *et al.* 2012; Argiriou *et al.* 2012; Tuttle *et al.* 2015). Genome-scale surveys have elucidated many of the genes that are differentially expressed between wild and domesticated cotton (Hovav *et al.* 2008b; Chaudhary *et al.* 2009; Rapp *et al.* 2010; Yoo and Wendel 2014; Nigam *et al.* 2014), or among developmental stages of fiber development (Shi *et al.* 2006; Gou *et al.* 2007; Taliercio and Boykin 2007; Hovav *et al.* 2008c, 2008b; Al-Ghazi *et al.* 2009; Rapp *et al.* 2010; Wang *et al.* 2010; Yoo and Wendel 2014; Nigam *et al.* 2014; Tuttle *et al.* 2015). These many studies indicate that domestication has dramatically altered the transcriptome of cotton fiber development, but to date the specific upstream variants and interacting partners responsible for these downstream developmental differences remain to be discovered.

From a genetic perspective, multiple independent quantitative trait loci (QTL) analyses have been performed to identify chromosomal regions contributing to phenotypic variation among various cotton genotypes. Most QTL analyses to date have focused either on crosses between modern cultivars of *G. hirsutum* or on crosses between cultivated forms of *G. hirsutum* with *G. barbadense*, another cultivated species which possesses superior fiber quality but with the limitations of lower yield and a narrower range of adaptation (Fang *et al.* 2017c; Chandnani *et al.* 2017; Hu *et al.* 2019). Interspecific cotton crosses often generate negative genetic correlations between fiber quality and lint yield, and these frequently suffer from F_2_ breakdown (reviewed in (Zhang *et al.* 2014)). Taken together, these numerous studies have reported more than 2,274 QTL (Said *et al.* 2015a) pertaining to agronomically and economically important traits (*e.g.*, plant architecture; biotic and abiotic stress resistance; fiber, boll, and seed quality and productivity). Several meta-analyses have attempted to identify possible QTL clusters and hotspots by uniting these QTL studies through a consensus map (Rong *et al.* 2007; Lacape *et al.* 2010; Said *et al.* 2015b, 2015a); QTL clusters denote genomic regions containing myriad QTL, whereas QTL hotspots are clusters of QTL for a single trait (Said *et al.* 2015b). These meta-analyses compiled QTL studies of both intraspecific *G. hirsutum* populations and interspecific *G. hirsutum* × *G. barbadense* populations, ultimately creating a QTL database from intraspecific and interspecific populations (Said *et al.* 2015a). To date, QTL analyses have yielded multiple, sometimes conflicting, insights that are accession- or environment-dependent. Some aspects of fiber development, for example, are associated with QTL enrichment in the D-subgenome of polyploid cotton (Jiang *et al.* 1998; Lacape *et al.* 2005; Han *et al.* 2006; Rong *et al.* 2007; Qin *et al.* 2008; Said *et al.* 2015b), which derives from a short fibered ancestor, but not all mapping populations reflect this bias (Ulloa *et al.* 2005; Lacape *et al.* 2010; Li *et al.* 2013). Likewise, QTL found in some environments and/or populations are not significant in similar, but non-identical, environments or in other mapping populations (Lacape *et al.* 2010; Said *et al.* 2015b, 2015a). Some data suggests that cotton fiber QTL are genomically clustered, yet with heterogeneous phenotypic effects (Rong *et al.* 2007; Qin *et al.* 2008; Lacape *et al.* 2010). Said *et al.* (Said *et al.* 2013, 2015b) showed that just as QTL clusters and hotspots exist for fiber quality, they also exist for other traits (*e.g.*, yield, seed quality, leaf morphology, disease resistance), and these hotspots, while found on every chromosome, tend to concentrate in specific regions of the genome. In particular, comparisons between intraspecific and interspecific populations reveal common QTL clusters and hotspots, possibly indicative of shared genetic architecture among cultivars and between species (Said *et al.* 2015b). While these QTL analyses have increased our understanding of the number and location of chromosomal regions that contribute to differences between cultivars and species, there remains a significant gap in our understanding of genes targeted during the initial domestication of cotton and their effects, which ultimately led to the development of modern cultivars.

Here we provide an evolutionary quantitative genetics perspective on the domestication of the dominant cultivated cotton species, *G. hirsutum*, through identification and characterization of QTL for traits that have played important roles during domestication. In contrast to previous studies, we utilize an *intraspecific* cross between a truly wild form of *G. hirsutum* (var. *yucatanense*, accession TX2094) and an elite cultivar (*G. hirsutum* cv. Acala Maxxa), to bracket the “before” and “after” phenotypic characteristics of the domestication process that played out over the last 5,000 years or so. Numerous domestication-related traits were characterized in both the parents and their segregating progeny in two environments, representing characters from several broader phenotypic categories: (1) plant architecture, (2) fruiting habit, (3) phenology, (4) flower, (5) seed, (6) fiber-length, (7) fiber quality, and (8) fiber color. We generated a SNP-based genetic linkage map to anchor each QTL to the *G. hirsutum* cotton reference genome (elite accession TM1; (Yu *et al.* 2013; Saski *et al.* 2017)) and identify plausible candidate genes for each trait. We show that the QTL associated with *G. hirsutum* domestication are both clustered and environmentally labile. Possible candidate genes were recovered and discussed for each trait. This study provides valuable insights into the genetic basis of cotton domestication and provides information that will assist in identifying cotton domestication genes and their functional effects on cotton biology.

## Materials and Methods

### Plant materials and phenotyping

A total of 466 F_2_ individuals were derived from a cross between *Gossypium hirsutum* var. *yucatanense* accession TX2094 as the maternal parent (USDA GRIN accession PI 501501, collected by J. McD. Stewart) and the modern elite cultivar *G. hirsutum* cv. Acala Maxxa as the paternal parent. The *G. hirsutum* var. *yucatanense* accession was previously identified as being truly wild using both allozyme (Wendel and Albert 1992) and RFLP analysis (Brubaker and Wendel 1994), as well as by morphological evidence. To allow for the replication of alleles over time and space, these individuals were grown as two subpopulations (October 2009 to July 2010), with 232 plants located in a greenhouse at Iowa State University (Ames, Iowa), and the remaining 234 in a greenhouse at the U. S. Arid-Land Agricultural Research Center (Maricopa, Arizona); nine representatives of each parental accession were also grown in each greenhouse. At Iowa State, individual seeds were separately planted in 7.6 L (two gallon) containers containing 15:7:3:3 soil:sand:peat:perlite. Plants were grown under natural sunlight (10-11 hr of daylight) with daytime and nighttime temperatures of 25 ± 2 and 20 ± 2°, respectively. Plants were fertilized twice a week with 125 ppm N. In Arizona, individual seeds were separately planted into 18.9 L (five gallon) pots containing moistened Sunshine Mix #1 (Sun Gro Horticulture Inc., Bellevue, WA) and perlite (4:1 ratio). Plants were grown under natural sunlight in a greenhouse with daytime and nighttime temperatures at 30 ± 2 and 22 ± 2°, respectively. All Arizona, plants were fertilized every two-weeks with 20–20–20 (200 ppm N) Peters Professional plant nutrient solution. These two populations were subsequently evaluated for multiple traits in each of the following eight categories: (1) plant architecture, (2) fruiting habit, (3) phenology, (4) flower, (5) seed, (6) fiber length, (7) fiber quality, and (8) fiber color (Table 1). Traits were selected to cover the range of possible domestication phenotypes.

**Table 1:** List of domestication-related traits measured in this study. For detailed information on identified QTL, refer to Table 2

Category	Trait
Plant architecture (10)	Plant Height (PH; mm); Fruiting Branch Length for 1^st^, 3^rd^ and 5^th^ branches (FB1, FB2, FB3; mm); Plant Height-to-Fruiting Branch Length Ratio (PHFB1, PHFB2, PHFB3); Branch Angle of 5^th^ Sympodium (BA; °); Node with Red Branch*^a^*; Average Stem Pubescence (SP)
Fruiting habit (7)	Total Number of Nodes (TN); Plant Height-to-Total Number of Nodes Ratio (PHTN); Total Number of Nodes to First Fruiting Branch (NF); Total Number of Non-Fruiting Branches (TNFB); Total Number of Fruiting Branches (TFB); Total Number of Newly Produced Nodes during 30-day Interval*^a^*; Total Number of Fruiting Branches after 30-day Interval*^a^*
Phenology (10)	Days to First Flower (FF); Total Number of Nodes at FF (TNFF)*^a^*; Total Number of Nodes to Fruiting Branch at FF*^a^*; Total Number of Fruiting Branches at FF*^a^* (FBFF); Total Number of Flowers during 30-day Interval; Average Number of Flowers/Day; Total Number of Open Bolls Retained after 30 Days + 4 Week Interval*^b^*; Total Number of Green Bolls Retained after 30 Days + 4 Week Interval (GB); Total Number of Bolls at 1^st^ Day of 30-day Interval (NB)*^a^*; Total number of Bolls at 30^th^ Day of 30-day Interval*^a^*
Flower (4)	Pollen Color (PC; Yellow/Cream); Petal Spot (PS; Presence/Absence); Average Stigma Distance (SD; mm); Curly Style (CS; Presence/Absence)*^a^*
Seed (7)	50 Fuzzy Seed Weight (FSW; g); 50 Seed Weight (SW; g); Average Number of Mature Seeds (5 Bolls); Average Seeded Cotton Weight (SCW; g; 5 Bolls); Average Number of Locules (AL; 5 Bolls); Average Boll Weight (BW; g; 5 Bolls)*^a^*; Average Weight of Locules (g; 5 Bolls)*^a^*
Fiber length (7)	Mean Length by Number (Ln; in); Coefficient of Variation of the Length by Number (LnCV; %); Mean Length by Weight (Lw; in); Coefficient of Variation of the Length by Weight (LwCV; %); 2.5% Length by Number (L25n; %; in); 5% Length by Number (L5n; %; in); Upper Quantile Length by Weight (UQLw; in)
Fiber color (3)	mean L* (CL), mean a* (Ca), mean b* (Cb)
Other fiber qualities (14)	Number of Dust Particles per g (Dust Count by g); Fineness (Fine; mTex); Immature Fiber Content (IFC; %); Maturity Ratio (MR); Nep Size (NS; μm); Neps per g; Seed Coat Nep Size (SCN Size; μm); Seed Coat Nep Count per g (SCN Count by g); Short Fiber Content by Number (SFCn; %); Short Fiber Content by Weight (SFCw; %); Total Count per g; Number of Trash Particles per g (Trash Count by g); Trash Size (TrS; μm); Visible Foreign Matter (VFM; %)

L* is a lightness component, ranging from 0 to 100 (from dark to bright), and a* (from green to red) and b* (from blue to yellow) are chromatic components ranging from -120 to 120 (Yam and Papadakis 2004)

a Traits were measured in Iowa subpopulation only.

b Traits were measured in Arizona subpopulation only.

At 150 (±7) days after planting, 10 plant architecture traits were evaluated, which include plant height, fruiting branch length, branch angle, and stem pubescence (Table 1). Data were collected for branch angles at the intersection of 1^st^, 3^rd^ and 5^th^ sympodia (secondary axes) with the main stem; however, due to high variation in the data observed from the 1^st^ and 3^rd^ sympodia, only data from the 5^th^ sympodium was considered further. In addition, the first node having a branch with red coloring was recorded in the Iowa population only (Table 1). Stem pubescence was scored independently by two people using the five-grade (1–5) ordinal scale developed by Lee (1968) (Lee 1968), where 1 is fully pubescent; the average of the two scores was recorded.

Traits relating to phenology, flowering, and fruiting were also examined. Eleven phenological traits (Table 1) were recorded, and, for consistency between the two greenhouse subpopulations, we hand-pollinated flowers for 30 days following the emergence of the first flower. Four floral traits were examined, including pollen color, the presence or absence of petal spot, average stigma distance (mm), and the presence or absence of curly styles. For pollen color, there exists a gradient of color from cream to yellow; however, we restricted our classifications to the parental color codes, *i.e.*, “cream” *vs.* “yellow” observed in Acala Maxxa and TX2094, respectively. Upon maturation, seven traits related to boll/seed development were also measured on harvested bolls, such as number of mature seeds, fuzzy seed weight, and average seeded cotton weight (Table 1).

Finally, 358 fiber samples harvested from the 466 F_2_ plants were collected and sent to the Cotton Incorporated Textile Services Laboratory (Cotton Incorporated, Cary, NC) for analysis by the AFIS Pro system (Uster Technologies, Charlotte, NC), an industry standard for evaluating fiber length and other quality traits (Table 1). Fiber color was determined by a MiniScan XE Plus colorimeter (ver. 6.4, Hunter Associates Laboratory, Inc., Reston, VA), which measures color properties of L*, a*, and b*. L* is a lightness component, ranging from 0 to 100 (from dark to bright), while a* (from green to red) and b* (from blue to yellow) are chromatic components ranging from -120 to 120 (Yam and Papadakis 2004). Values were measured three times on the same fiber sample and averaged for each trait (*i.e.*, mean L*, mean a*, and mean b*).

### Genotyping and genetic map construction

A total of 384 KASPar-based SNP assays (277 co-dominant) were used to genotype the 466 F_2_ plants with phenotypic data (KBioscience Ltd., Hoddesdon, UK). SNP assays were designed as previously reported for *G. hirsutum* (Byers *et al.* 2012). Genomic DNA was extracted from leaf tissue using the Qiagen DNeasy Plant Mini Kit (Qiagen, Stanford, CA, USA) and normalized to an approximate concentration of 60 ng/µL.

Specific target amplification (STA) PCR was used to pre-amplify the target region of genomic DNA containing the SNPs of interest, but without the discriminating SNP base in the primer sequence. The PCR conditions for this protocol included a 15-min denaturing period at 95° followed by 14 two-step cycles: 15 s at 95° followed by 4 min at 60°. This effectively increased the concentration of the target DNA relative to the remaining DNA. The sample amplicons produced by the STA protocol were then genotyped using the Fluidigm 96.96 Dynamic Arrays genotyping EP1 System (San Francisco, CA). Each Fluidigm plate run included eight control samples: two Acala Maxxa, two TX2094, two pooled parental DNA (synthetic heterozygotes), and two no-template controls (NTC). These controls served as guideposts during the genotyping process. The STA amplicons and the SNP assays were loaded onto a Fluidigm 96.96 chip, where a touchdown PCR protocol on the Fluidigm FC1 thermal cycler (San Francisco, CA, USA) was used to allow the competing KASPar primers to amplify the appropriate SNP allele in each sample.

Fluorescence intensity for each sample was measured with the EP1 reader (Fluidigm Corp, San Francisco, CA) and plotted on two axes. Some assays required more amplification in order to produce distinct clusters. For those that did not form distinct clusters during the initial analysis, an additional five cycles of PCR were performed on the plate and fluorescence intensity measured again until all assays produced sufficient resolution for cluster calling. Genotypic calls based on EP1 measurements were made using the Fluidigm SNP Genotyping Analysis program (Fluidigm 2011). All genotype calls were manually checked for accuracy and ambiguous data points that either failed to amplify and/or cluster near parental controls were scored as missing data. The final raw output for an individual chip included data from each of the multiple scans performed to ensure that the optimal amplification conditions for each assay was represented. The text output from genotyping was arranged to a compatible format for genetic mapping using Excel. Files are available at https://github.com/Wendellab/QTL_TxMx.

A genetic linkage map based on the KASPar genotyping data were constructed separately for each subpopulation using regression mapping as implemented in JoinMap4 (Van Ooijen 2011). A LOD threshold of 5.0 was used and linkage distances were corrected with the Kosambi mapping function. Loci were excluded from the map if they failed to meet a Chi-Square test (α = 0.05) for expected Mendelian ratios. Separate linkage maps (*i.e.*, not a single composite linkage map) were used for QTL analysis in each subpopulation to maximize independence when comparing results between Iowa and Arizona.

### QTL analysis

**Figure 2:**
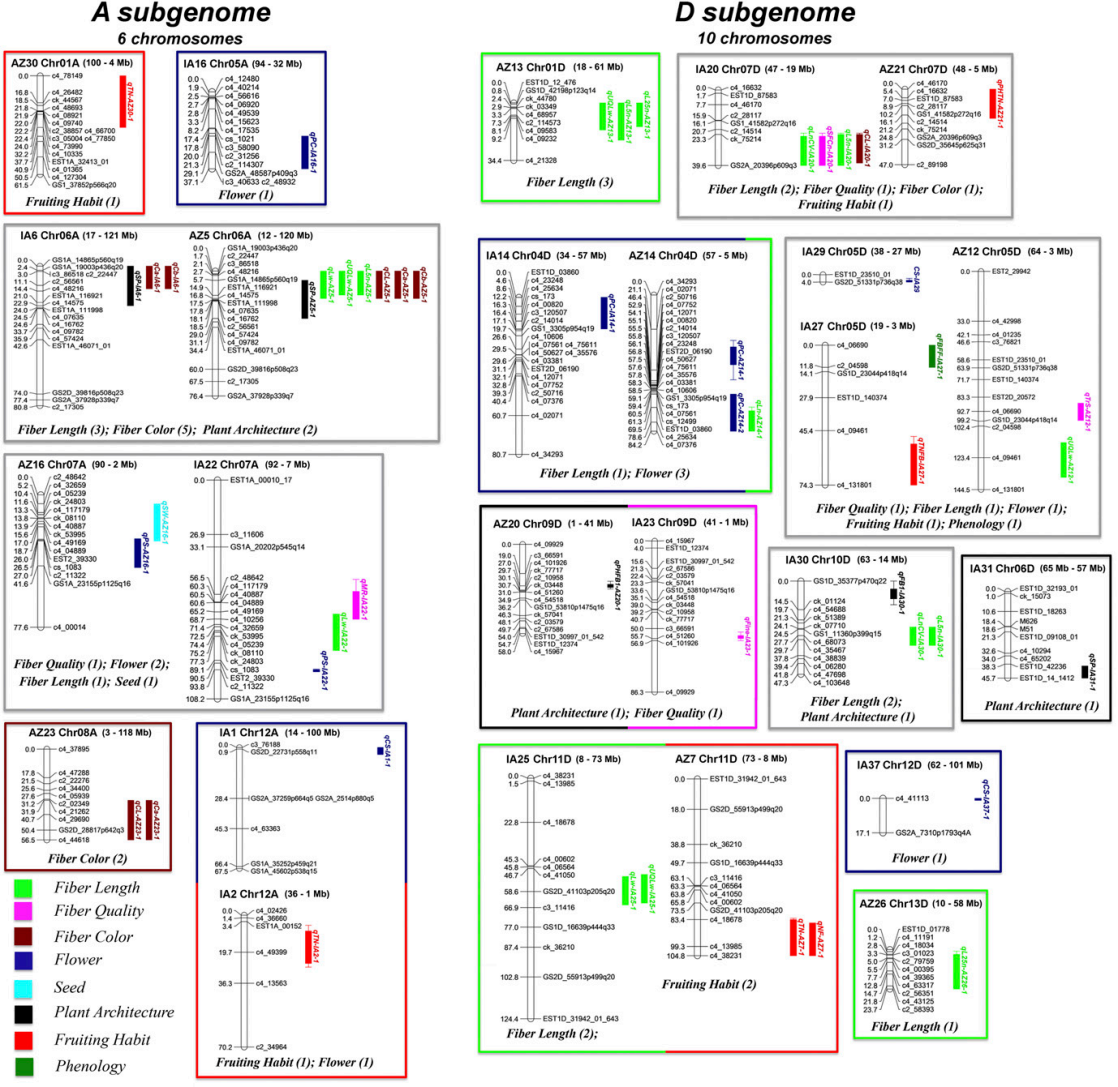
Genetic linkage map that includes the top 50 QTL associated with cotton domestication traits evaluated here, as generated by MapChart 2.2 (Voorrips 2002). While all chromosomes were recovered for the linkage map, only those linkage groups/chromosomes containing QTL are depicted here. QTL nomenclature follows that first used in rice (McCouch *et al.* 1997), which starts with “q”, followed by an abbreviation of the trait name. Environments are designated at the end of the QTL name with “AZ” (Arizona) or “IA” (Iowa). QTL are colored by trait category. Confidence intervals for QTL are plotted as one-LOD interval. Genomic ranges for each LG are specified. For specific locations on the *G. hirsutum* genome sequence, LOD scores, and other details, see Table 3 and Supplemental Table 2.

For each location, the raw phenotypic values of each trait were evaluated for statistical outliers in SAS version 9.3 (SAS Institute 2012) by examination of Studentized deleted residuals (Kutner *et al.* 2004), which were obtained from a simple linear model fitted with fixed effects for the grand mean and a single randomly sampled, representative SNP marker. QTL were detected within each greenhouse environment (Ames, IA and Maricopa, AZ) with Windows QTL Cartographer V2.5 (Wang *et al.* 2012) using the composite interval mapping (CIM) method (Zeng 1993, 1994) with a window size of 10 cM and a 1 cM walk speed. The LOD thresholds used to identify QTL were determined using a permutation test (1000 repetitions, α = 0.05) (Churchill and Doerge 1994), and the confidence intervals were set as the map interval corresponding to one-LOD interval on either side of the LOD peak (Mangin *et al.* 1994). If the QTL were separated by a minimum distance of 20 cM, they were considered two different QTL (Ungerer *et al.* 2002). To identify coincident QTL between subpopulations for each trait, we determined whether SNP markers were shared between QTL intervals. If at least one marker was shared between QTL marker intervals, then we concluded that the same QTL (*i.e.*, coincident QTL) was identified in both subpopulations. A QTL cluster was declared where three or more QTL of different trait categories occurred within a 20 cM region, and a QTL hotspot was declared where three or more QTL of the same trait category occurred within a 20 cM region following (Said *et al.* 2015b) with modification for a single genetic cross. Both QTL clusters and QTL hotspots were declared within each subpopulation, but coincident QTL clusters and QTL hotspots between subpopulations were only counted once with respect to the total of each QTL class. The linkage map showing the location of QTL (Figure 2) was generated by MapChart 2.2 (Voorrips 2002) and colorized in Adobe Photoshop Creative Suite 5 (Adobe). QTL nomenclature follows a method used in rice (McCouch *et al.* 1997), which starts with “q”, followed by an abbreviation of the trait name. The population from which the QTL derived is abbreviated at the end as “AZ” and “IA”, for Arizona and Iowa, respectively.

### Candidate gene searches

Linkage groups were assigned to *G. hirsutum* chromosomes (Table 2) using molecular marker sequences as gmap (Wu and Watanabe 2005; Wu and Nacu 2010) queries against the published *G. hirsutum* cv TM-1 (CottonGen Download TM-1; Saski *et al.* 2017) genome (annotation gff version 1.1), using default values and permitting two possible paths (to accommodate homeologs). A consensus of markers was used to identify the candidate chromosome for each linkage group, using the highest scoring path for each marker; however, when both paths were equally likely, both were used to derive the consensus. Candidate genes contained within the QTL confidence interval were identified by using the genomic coordinates of the first and last marker for each linkage group as a boundary, and subsequently intersecting the genomic boundaries of each linkage group with the genome annotation via bedtools 2 (Quinlan and Hall 2010). Orthogroups between the *G. hirsutum* genome used here and other published cotton genomes were generated via Orthofinder (Emms and Kelly 2015, 2019). Orthogroup results are not reported, but are provided for reference in Supplemental File 1. All scripts and parameters are available at https://github.com/Wendellab/QTL_TxMx.

**Table 2:** Subgenome location of linkage group based on linkage map and genomically mapped markers. The number of markers used to identify the chromosomes is listed. Start and end show the position in the corresponding *G. hirsutum* cv. TM-1 subgenome

Linkage group (AZ)	Linkage group (IA)	*G. hirsutum*^a^**	start	end	*G. arboreum*	*G. raimondii*
AZ30	IA24	ChrA01	4,271,138	100,276,588	Chr01/Chr02	
AZ25		ChrA02	326,615	84,855,696	Chr03	
	IA11	ChrA02	3,870,558	84,855,696	Chr03	
	IA12	ChrA02	326,615	1,008,410	Chr03	
AZ10	IA07	ChrA03	7,756,446	101,464,731	Chr03	
AZ33	IA32	ChrA04	807,278	75,497,922	Chr06	
AZ06	IA16	ChrA05	32,455,072	93,933,072	Chr05	
AZ11	IA34	ChrA05	12,447,798	17,185,964	Chr05	
AZ05	IA06	ChrA06	11,844,977	121,378,180	Chr06	
AZ16		ChrA07	1,830,647	89,848,877	Chr06	
AZ17		ChrA07	92,681,306	93,171,853	Chr07	
	IA22	ChrA07	7,321,899	93,171,853	Chr07	
AZ23	IA19	ChrA08	2,877,637	117,527,721	Chr08	
AZ24	IA05	ChrA09	2,580,082 (15,659,999)	79,333,397 (75,848,634)	Chr09	
AZ19	IA15	ChrA10	6,056,379 (6,566,496)	106,114,506	Chr10	
AZ08	IA26	ChrA11	1,912,510	4,371,131	Chr11	
AZ15		ChrA11	10,951,928	109,621,794	Chr11	
	IA17	ChrA11	53,172,447	103,552,230	Chr11	
	IA18	ChrA11	10,951,928	12,955,059	Chr11	
AZ01	IA02	ChrA12	785,478	78,273,367 (72,842,063)	Chr12	
AZ03	IA01	ChrA12	77,411,923 (13,521,801)	100,079,948	Chr12	
AZ18	IA08	ChrA13	3,404,007	96,773,239	Chr13	
AZ13	IA10	ChrD01	18,196,452	62,287,774		Chr02
AZ27	IA33	ChrD02	12,742,894	61,010,129		Chr05
AZ28	IA36	ChrD03	6,483,364	50,172,131 (48,393,682)		Chr03
AZ14	IA14	ChrD04	3,602,330	56,438,319		Chr12
AZ12		ChrD05	2,523,538	63,761,721		Chr09
	IA27	ChrD05	2,523,538	18,861,200		Chr09
	IA28	ChrD05	32,622,237	63,761,721		Chr09
	IA29	ChrD05	26,606,552	27,776,136		Chr09
AZ31	IA31	ChrD06	57,362,695	65,851,264		Chr10
AZ21	IA20	ChrD07	5,155,281 (18,304,091)	48,192,327		Chr01
AZ22	IA21	ChrD07	55,033,970	55,696,530		Chr01
AZ09	IA04	ChrD08	2,309,559 (4,206,266)	69,750,855		Chr04
AZ20	IA23	ChrD09	1,234,789	40,676,126		Chr06
AZ32	IA30	ChrD10	13,976,894	62,550,932		Chr11
AZ07	IA25	ChrD11	7,839,868	72,873,302		Chr07
AZ02	IA03	ChrD12	22,239,698	53,411,834 (51,612,631)		Chr08
AZ04	IA37	ChrD12	61,838,133	101,355,435		Chr08
AZ26	IA09	ChrD13	8,757,166	58,413,467		Chr13
AZ29	IA13	ChrD13	62,947,661			Chr13
AZ34	IA35	ChrD13	852,543	1,182,162		Chr05

a https://www.cottongen.org/species/Gossypium_hirsutum/jgi-AD1_genome_v1.1

Candidate genes were further screened for previously established expression differences in developing fibers (Bao, Hu *et al.*, 2019), for putative transcription factors (CottonGen Download TM-1; Saski *et al.* 2017), and for non-silent SNPs between the parental accessions. For the latter, reads derived from *G. hirsutum* Acala Maxxa (SRA:SRR617482) and *G. hirsutum* TX2094 (SRA:SRR3560138-3560140) were mapped against the TM-1 genome (CottonGen Download TM-1; Saski *et al.* 2017) and SNPs were annotated using the Best Practices pipeline of GATK (Van der Auwera *et al.* 2013). The resulting vcf files were processed with vcftools (Danecek *et al.* 2011) and SnpSift (Cingolani *et al.* 2012a) to (1) only recover sites with differences between *G. hirsutum* Acala Maxxa and *G. hirsutum* TX2094, (2) remove sites with missing data, and (3) only recover SNPs where the wild *G. hirsutum* TX2094 shared the ancestral SNP with an outgroup species, *G. mustelinum* (SRA: SRR6334743). The resulting 3.6 million SNPs were annotated with SnpEff (Cingolani *et al.* 2012b) for the putative effects of each change, and SnpSift was again used to restrict the final vcf to only those SNPs where an effect was annotated. In addition, previously identified selective sweeps found in another *G. hirsutum* cv TM1 genome version (Fang *et al.* 2017a; Wang *et al.* 2017b) were placed on the *G. hirsutum* cv TM1 used here by comparing the genomes with MUMMER (Marçais *et al.* 2018) and intersecting coordinates with bedtools2 (Quinlan 2014). The final set of genes with annotated effects was further limited to only those regions under a QTL. These genes were additionally classified as to whether they also: (1) exhibit differential expression; (2) are putative TFs; or (3) belong to a curated list of potentially fiber-relevant cotton genes, based on existing literature (Fang 2018). Putative functional annotations were downloaded from CottonGen. The QTL peak was placed on the genome sequence by using the genomic QTL boundaries (determined above) to relate the number of cM to the amount of sequence in that same region (in base pairs). All program run information and relevant parameters are available at https://github.com/Wendellab/QTL_TxMx.

### Data availability

All data and scripts are available via GitHub (https://github.com/Wendellab/QTL_TxMx). All other data, *e.g.*, genomes and downloaded sequences are listed in the methods. Seed from the mapping population is available from the GRIN National Genetic Resources Program. Supplemental material available at figshare: https://doi.org/10.25387/g3.10304945.

## Results

### Phenotypic variation

Most traits investigated (Table 1) exhibited phenotypic variability between two parents, TX2094 and Acala Maxxa (Supplemental Table 1). In general, the phenotypes reflected the expected “domestication syndrome” in Acala Maxxa, as represented by its: (1) reduced plant height; (2) fewer total nodes; (3) fewer nodes to first fruiting branch; (4) better fruiting habit (*e.g.*, longer fruiting branches); (5) early flowering; (6) greater production of flowers, bolls, and seeds; and (7) enhanced fiber quantity and quality (Supplemental Table 1). The F_2_ plants displayed a wide range of phenotypic variability in two greenhouse environments, Ames, IA, and Maricopa, AZ. The northern latitude of Iowa contributed to variability for traits reflective of a cooler, less-sunny environment compared to the F_2_ plants grown in Arizona. That is, plants grown in Iowa typically were taller, with shorter fruiting branch lengths and a greater number of nodes; however, these plants also exhibited a greater number of nodes to first fruiting branch, as well as a higher ratio of non-fruiting to fruiting branches. Interestingly, the Iowa subpopulation also exhibited both later flowering and more flowers during a 30-day interval. The flowers themselves exhibited greater distance between stigma and style, and produced more seeds per boll with an overall lighter seed weight (per boll), indicative of smaller seed size. Other flower and fiber traits exhibited continuous variation in all the F_2_ plants, from TX2094-like to Acala Maxxa-like phenotypes; however, the two subpopulations were often statistically distinguishable. For example, 50 Fuzzy Seed weight (g) was 3.96 and 4.13 in Iowa and Arizona, respectively, which is significantly different (α *=* 0.05). Observations such as these are unexpected under the null hypothesis that subpopulations should not be phenotypically distinct, and they likely reflect an interaction with the environment. Phenotypic measurements for parents and progeny are found in Supplemental Table 1.

### Linkage map construction

KASPar-based SNP genotyping was used to construct separate genetic linkage maps (total genetic length of 1704.03 cM for the Arizona subpopulation and 1989.46 cM for the Iowa subpopulation) from the *G. hirsutum* F_2_ subpopulations using JoinMap (Stam 1993). Of the 384 markers used for genotyping, 356 were successfully mapped to create 34 linkage groups for the Arizona population, and 336 were mapped to create 37 linkage groups for the Iowa population (Table 2). Among those 384 originally targeted markers, 84 markers were homeolog-specific by design (see Byers *et al.* 2012). To determine whether the homeologous genome of these markers was specific and accurately identified, linkage groups with multiple homeolog-diagnostic SNPs were examined for genome consensus. Seventy (83%) of the 84 assays resided in linkage groups with at least one other homeologous assay. The homeologous genome assignment for these linkage groups was consistent with the genome sequence and the candidate gene/chromosome identification (see below). These linkage groups cover all 26 chromosomes in the *G. hirsutum* genome (Table 2).

### Identification of QTL and QTL clusters

A total of 120 QTL were detected from marker-trait analysis of the two subpopulations (Figure 2, Supplemental Table 2). The QTL detected from the subpopulations represented all phenotypic categories (53 QTL for 28 traits in the Iowa population; 67 QTL for 29 traits in the Arizona population). These QTL map to 22 and 24 linkage groups (20 and 21 chromosomes) in the Arizona and Iowa subpopulations, respectively; 59 QTL mapped to 12 chromosomes of A_T_ subgenome, while 61 QTL mapped to 12 chromosomes of D_T_ subgenome (Supplemental Table 2). In general, these *G. hirsutum* chromosomes carry a mean and median of 5 and 5.5 QTL respectively; however, three chromosomes (A02, A09 and A13) have only a single QTL each and two (A06, A07) include 10 QTL each (Supplemental Table 2). Combining QTL mapping results from two subpopulations, 11 QTL clusters were identified for 23 traits in eight trait categories (Supplemental Table 2). Seven QTL hotspots were identified on chromosomes A06 and A08 for fiber color, and chromosomes A6, A7, D01, D04 and D13 for fiber length (Supplemental Table 2). The top 50 QTL (R^2^ > 10%) are summarized in Table 3. A full listing of identified QTL, map, and genomic information, and other relevant information is included in Supplemental Tables 2 and 3, and is discussed in the context of phenotype (see below).

**Table 3:** Top 50 QTL associated with domestication traits. For full list of QTL, see Supplemental Table 2

Category	Trait*^a^*	Chr*^b^*	QTL name*^c^*	Marker interval	Peak position (cM)	Peak position (Mb)*^d^*	LOD	*A**^e^*	*D**^f^*	|d/a|*^g^*	GA*^h^*	R^2^(%)*^i^*
Fruiting habit	TN	A01	qTN-AZ30-1	c4_78149-EST1A_32413_01	22.20	65.15	8.68	−2.28	−0.97	0.42	PD	12.68
Flower	PC	A05	qPC-IA16-1	c2_114307-c2_48932	36.07	32.46	8.13	−0.11	0.11	1.02	D	13.82
Fiber color	Ca	A06	qCa-AZ5-1	GS1A_19003p436q20-EST1A_111998	6.72	17.16	90.69	−2.27	0.15	0.07	A	75.47
Fiber color	Ca	A06	qCa-IA6-1	GS1A_14865p560q19-c4_48216	1.01	17.16	66.58	7.63	−1.63	0.21	PD	75.40
Fiber color	Cb	A06	qCb-AZ5-1	GS1A_19003p436q20-EST1A_111998	6.72	17.16	99.53	−5.22	1.22	0.23	PD	79.89
Fiber color	Cb	A06	qCb-IA6-1	GS1A_14865p560q19-c4_48216	1.01	17.16	55.90	−2.23	0.45	0.20	PD	43.81
Fiber color	CL	A06	qCL-AZ5-1	GS1A_19003p436q20-EST1A_111998	6.72	17.16	59.81	6.76	−0.39	0.06	A	65.20
Fiber length	L5n	A06	qL5n-AZ5-1	GS1A_19003p436q20-EST1A_116921	5.72	17.16	6.36	0.03	0.04	1.06	D	12.14
Fiber length	Lw	A06	qLw-AZ5-1	GS1A_19003p436q20-EST1A_111998	5.72	17.16	6.23	0.03	0.02	0.68	PD	11.66
Plant architecture	SP	A06	qSP-AZ5-1	GS1A_14865p560q19-c4_09782	17.84	96.62	72.39	1.48	0.15	0.10	A	71.49
Plant architecture	SP	A06	qSP-IA6-1	GS1A_14865p560q19-EST1A_111998	11.14	100.61	43.49	1.20	0.02	0.01	A	48.49
Fiber length	UQLw	A06	qUQLw-AZ5-1	GS1A_19003p436q20-EST1A_116921	6.72	17.16	5.54	0.03	0.03	1.03	D	12.13
Fiber length	Lw	A07	qLw-IA22-1	c4_49169-cs_1083	69.75	72.79	5.43	−0.03	0.00	0.03	A	10.79
Other fiber qualities	MR	A07	qMR-IA22-1	GS1A_20202p545q14-c4_32659	67.17	72.79	4.13	0.66	1.45	2.18	OD	12.84
Flower	PS	A07	qPS-AZ16-1	EST2_39330-c4_00014	26.01	21.29	62.23	−0.38	0.51	1.33	OD	41.40
Flower	PS	A07	qPS-IA22-1	c2_11322-GS1A_23155p1125q16	93.82	18.38	37.42	−0.38	0.33	0.87	D	53.58
Seed	SW	A07	qSW-AZ16-1	c4_32659-GS1A_23155p1125q16	18.01	80.66	8.69	0.24	0.00	0.01	A	12.87
Fiber color	Ca	A08	qCa-AZ23-1	c4_21262-c4_44618	46.67	116.77	25.47	−0.87	0.02	0.02	A	12.93
Fiber color	CL	A08	qCL-AZ23-1	c4_21262-c4_44618	47.67	116.77	14.98	2.55	0.15	0.06	A	11.44
Flower	CS	A12	qCS-IA1-1	c3_76188-GS2A_37259p664q5	2.91	78.27	8.20	−0.26	−0.22	0.85	D	25.99
Fruiting habit	TN	A12	qTN-IA2-1	EST1A_00152-c4_13563	17.36	7.83	4.55	−1.40	1.97	1.41	OD	14.07
Fiber color	CL	D07	qCL-IA20-1	ck_75214-GS2A_20396p609q3	26.29	18.30	5.00	−0.03	0.00	0.07	A	12.28
Fiber length	L5n	D07	qL5n-IA20-1	ck_75214-GS2A_20396p609q3	28.29	18.30	4.49	2.65	1.65	0.62	PD	10.43
Fiber length	LnCV	D07	qLnCV-IA20-1	ck_75214-GS2A_20396p609q3	28.29	18.30	4.49	2.65	1.65	0.62	PD	10.43
Fruiting habit	PHTN	D07	qPHTN-AZ21-1	c4_46170-ck_75214	9.95	28.62	6.85	−3.41	−0.62	0.18	A	11.69
Other fiber qualities	SFCn	D07	qSFCn-IA20-1	ck_75214-GS2A_20396p609q3	29.29	18.30	4.62	2.64	1.60	0.61	PD	10.77
Fiber length	L25n	D01	qL25n-AZ13-1	EST1D_12_476-c4_21328	0.01	18.79*	5.33	−0.04	0.04	0.98	D	14.26
Fiber length	L5n	D01	qL5n-AZ13-1	EST1D_12_476-c4_21328	7.27	18.20	8.39	−0.05	0.00	0.05	A	14.27
Fiber length	UQLw	D01	qUQLw-AZ13-1	EST1D_12_476-c4_21328	6.27	18.20	5.77	−0.04	0.00	0.10	A	10.82
Other fiber qualities	Fine	D09	qFine-IA23-1	c3_66591-c4_101926	53.97	12.45	4.13	−1.50	−2.56	1.71	OD	14.59
Plant architecture	PHFB1	D09	qPHFB1-AZ20-1	c3_66591-ck_77717	26.97	38.62	5.51	15.64	−12.40	0.79	PD	10.49
Fiber length	Lw	D11	qLw-IA25-1	c4_41050-c3_11416	55.76	20.78	5.28	0.04	0.00	0.08	A	11.10
Fruiting habit	NF	D11	qNF-AZ7-1	c4_18678-c4_38231	101.27	10.42	7.53	−1.09	−0.76	0.70	PD	34.95
Fruiting habit	TN	D11	qTN-AZ7-1	c4_18678-c4_38231	104.27	10.42	4.38	−2.06	−0.70	0.34	PD	11.56
Fiber length	UQLw	D11	qUQLw-IA25-1	c4_41050-c3_11416	54.76	20.78	6.14	0.05	−0.01	0.29	PD	13.61
Flower	CS	D12	qCS-IA37-1	c4_41113-GS2A_7310p1793q4A	0.01	61.84	36.03	−0.43	−0.55	1.28	OD	66.09
Flower	CS	D05	qCS-IA29-1	EST1D_23510_01-GS2D_51331p736q38	3.01	27.78	30.54	0.41	−0.54	1.31	OD	64.96
Phenology	FBFF	D05	qFBFF-IA27-1	c4_06690-GS1D_23044p418q14	0.01	18.86	4.79	−2.08	−2.07	1.00	D	14.85
Fruiting habit	TNFB	D05	qTNFB-IA27-1	c4_09461-c4_131801	61.46	9.09	4.33	−1.32	−1.03	0.79	PD	10.31
Other fiber qualities	TrS	D05	qTrS-AZ12-1	EST2D_20572-GS1D_23044p418q14	92.71	18.86	5.11	19.84	−7.19	0.36	PD	14.06
Fiber length	UQLw	D05	qUQLw-AZ12-1	c2_04598-c4_131801	125.40	9.09	4.71	−0.03	0.05	1.54	OD	17.51
Plant architecture	SP	D06	qSP-IA31-1	EST1D_42236-EST1D_14_1412	45.29	55.33*	14.64	0.61	0.20	0.33	PD	12.47
Plant architecture	FB1	D10	qFB1-IA30-1	GS1D_35377p470q22-ck_01124	6.01	62.55	4.05	0.13	1.26	9.64	OD	12.33
Fiber length	L5n	D10	qL5n-IA30-1	ck_51389-c4_38839	26.55	13.98	5.15	2.34	−2.69	1.15	D	11.51
Fiber length	LnCV	D10	qLnCV-IA30-1	ck_51389-c4_38839	26.55	13.98	5.15	2.34	−2.69	1.15	D	11.51
Fiber length	Ln	D04	qLn-AZ14-1	EST1D_03860-c4_07376	80.59	3.60	4.40	−0.03	−0.01	0.33	PD	10.93
Flower	PC	D04	qPC-AZ14-1	c4_02071-c2_50716	37.10	46.08*	2.77	−0.10	0.10	0.99	D	10.58
Flower	PC	D04	qPC-AZ14-2	cs_12499-c4_07376	80.59	3.60	11.48	−0.12	0.14	1.13	D	20.11
Flower	PC	D04	qPC-IA14-1	EST1D_03860-c4_00820	10.61	39.70	7.73	−0.10	0.13	1.28	OD	13.90
Fiber length	L25n	D13	qL25n-AZ26-1	c4_18034-c2_58393	19.68	58.41	3.88	0.05	0.00	0.08	A	10.76

a Fiber color: Ca, mean a*; Cb, mean b*; CL, mean L*; Fiber length: L25n, 2.5% Length by Number; L5n, 5% Length by Number; Ln, Mean Length by Number; LnCV, Coefficient of Variation of the Length by Number; Lw, Mean Length by Weight; UQLw, Upper Quantile Length by Weight; Flower: CS, Curly Style; PC, Pollen Color; PS, Petal Spot; Fruiting habit: NF, Total Number of Nodes to First Fruiting Branch; PHTN, Plant Height-to-Total Number of Nodes Ratio; TN, Total Number of Nodes; TNFB, Total Number of Non-Fruiting Branches; Other fiber qualities: Fine, Fineness; MR, Maturity Ratio; SFCn, Short Fiber Content by Number; TrS, Trash Size; Phenology: FBFF, Total Number of Fruiting Branches at First Flower; Plant architecture: FB1, Fruiting Branch Length for 1^st^ Branch; PHFB1, Ratio of PH to FB1; SP, Average Stem Pubescence; Seed: SW, 50 Fuzzy Seed Weight.

b Chromosome designation. A and D represents the A- and D- subgenome, respectively.

c QTL name is provided as follows: the first two to four letters excluding “q” indicate the abbreviated trait name, following by linkage group (LG). The last letter indicates the population in which the QTL was detected; IA, Iowa; AZ, Arizona.

d Positions marked with an * indicate estimates based on nearest genomically located markers.

e Additive (A) effect when substituting a TX2094 allele with an allele from Acala Maxxa at the QTL. The effect of the Acala Maxxa allele relative to the TX2094 allele at each QTL indicates the sign (positive or negative) of the allelic effect.

f Dominance (D) effect.

g |dominance effect/additive effect|

h Gene action. A, additive (|d/a| = 0-0.2); PD, partial dominance (|d/a| = 0.21-0.8); D, dominance (|d/a| = 0.81-1.2); OD, overdominance (|d/a| >1.2).

i Percentage of phenotypic variance explained by each QTL.

#### Connection of QTL to domestication:

Of the 120 QTL identified across the two subpopulations, Acala Maxxa had additive allelic effects that were positive (‘increasing allele’) or negative (‘decreasing allele’), relative to Tx2094, for 56 and 64 QTL, respectively (Supplemental Table 2). With respect to trait, Acala Maxxa had more positive effect alleles for the 14 QTL (10 positive *vs.* 4 negative effect alleles) and 16 QTL (14 positive *vs.* 2 negative effect alleles) associated with traits in the plant architecture and seed categories. In contrast, Acala Maxxa had more QTL with negative allelic effects for traits in the fruiting habit (3 positive *vs.* 9 negative), flower (2 positive *vs.* 15 negative), and phenology (1 positive *vs.* 6 negative) categories. Interestingly, Acala Maxxa exhibited a more balanced number of positive and negative allelic effect estimates for the fiber length (16 positive *vs.* 17 negative), fiber color (5 positive *vs.*, 8 negative), and other fiber qualities (5 positive *vs.* 3 negative). Collectively, these findings show that the QTL alleles contained within Acala Maxxa that associate with “domestication syndrome” attributes (*e.g.*, greater production of seed, reduced stature, increased fiber length) may influence the phenotype in a manner not readily apparent (*e.g.*, both positive and negative alleles associated with fiber length).

#### Candidate Gene identification:

A total of 28,531 genes (Supplemental Table 4) are predicted within the genomic range of the 120 QTL (Supplemental Table 2), representing approximately 42% of the predicted gene models for the *G. hirsutum* cv. TM1 genome (Saski *et al.* 2017). The genomic regions occupied by QTL average approximately 83 Mbp in size (median = 76 Mbp), for a total genomic length of approximately 1,353 Mbp or 60% of the total sequenced genome length of 2,260 Mbp (Supplemental Table 3). For each phenotype (*e.g.*, plant architecture, fiber color, etc), between 1,782- 11,807 distinct genes were recovered. Candidate genes for each phenotype are discussed below.

We further screened the 28,531 candidate genes for (1) genes with non-silent mutations in the domesticated Acala Maxxa (using the outgroup polyploid species *G. mustelinum* to infer the ancestral state), to filter for possible functional differences at the protein level; (2) genes with expression differences between Acala Maxxa and TX2094, to filter for genes that have been up- or down-regulated under domestication; (3) transcription factors; or (4) known cotton fiber genes of interest (see methods for details) (Supplemental Table 4). In general, fewer genes were found within the QTL boundaries for the A subgenome (13,185 *vs.* 15,346 in D_T_); while seemingly incongruent with the larger proportion of the A subgenome covered by QTL (approximately 847 Mbp in A_T_ *vs.* 506 in D_T_), this likely reflects gene density differences due to the twofold difference in subgenome size (A ∼2D).

From the genome-wide total of 34,870 genes that have one or more SNP between TX2094 and Acala Maxxa, 87% (30,337 genes) are affected by at least one putatively non-silent mutation. Over half of these genes have SNPs that change the amino acid (19,195 genes), and slightly more than half have changes in the untranslated regions (UTR; 19,829) in an approximately 3:5 ratio favoring mutations in the 5′ UTR. These are slightly greater than the number of genes that have silent SNPs (39%; 13,579 genes). Only 2.6% of genes have a SNP that changes the start or stop (in an approximate 2:3 ratio, start:stop). Genome-wide, there exists no bias toward the A or D subgenome for any of the above categories. Of those 30,337 genes with non-silent TX2094 *vs.* Acala Maxxa SNPs, 42% (12,744 genes) fall within a QTL in a ratio of approximately 0.8 A_T_:1 D_T_ (5,832 genes in A_T_ *vs.* 6,912 in D_T_). This ratio is approximately equivalent to the overall representation of the genome under QTL, *i.e.*, 0.9A_T_:1D_T_. Of the 12,744 genes with a non-silent SNP that occur under the QTL, 62% (7,925 genes) have predicted amino acid changes between TX2094 and Acala Maxxa (3,600 A_T_ genes and 4,325 D_T_) that could potentially be visible to selection (Table 4).

**Table 4:** Number of genes in any QTL, or for QTL related to a specific trait, that also exhibit additional differences between wild and domesticated cotton

	Total	Genes with non-silent changes *^a^*	Genes with non-synonymous changes	differentially expressed *^b^*	Transcription factors	Known cotton genes
All QTL	28,531	12,744	1,617	NA	176	42
Architecture	5,646	2,602	490	NA	32	6
Fiber Color	1,782	764	3247	144	11	5
Fiber Length	11,807	5,254	1,230	865	80	16
Other fiber qualities	4203	1,963	2370	342	30	3
Flower	8,272	3816	1472	NA	50	14
Fruiting Habit	5,136	2335	813	NA	31	6
Phenology	2,661	1,297	2409		17	1
Seed	9,116	3,929	921	NA	54	15

a includes start/stop adjustments and SNPs in UTR.

b DGE only applies to fiber-related traits.

To further explore the candidate genes under the QTL, we also quantified the number of genes under QTL that exhibit differential expression (DGE) during fiber development (Bao, Hu, *et al.* 2019). Of the 5,168 genes differentially expressed between TX2094 and Acala Maxxa (in either 10 or 20 dpa fiber; adjusted *P*-value < 0.005), approximately 42% (2,148, genes) are located under one of the QTL (Table 4), over half of which were located under a fiber QTL (1,147). Between 7–8% of genes for each phenotypic group experienced DGE in the fiber stages surveyed (10 and 20 dpa). Interestingly, there appears to be little bias toward differential expression of genes under fiber-related QTL *vs.* non-fiber QTL for these fiber-derived expression data. This may reflect a general overlap between fiber-relevant genes (*e.g.*, cell wall, cytoskeletal genes, etc) and those involved in broad plant phenotypes, as well as the remarkable increase in gene coregulation during domestication (Hu *et al.* 2016). Therefore, while we note differences in DGE for possible candidate genes from any trait category, the relevance of this fiber-derived DGE to non-fiber traits is unclear. Differentially expressed genes that also contain nonsynonymous and/or UTR SNPs account for about half of the DGE-QTL genes (1,137 genes), 723 of which have predicted amino acid changes.

Finally, we also considered two categories of genes of possible interest under the QTL: transcription factors (TF) and previously identified fiber-relevant genes (see methods). The QTL regions contained 176 putative TF (CottonGen Download TM-1; Saski *et al.* 2017) (74A:102D), representing approximately 1% of the genes related to each trait. Of these 176 TF, 97 had putative amino acid changes. Only three transcription factors under QTL exhibited expression changes, *i.e.*, Gohir.A04G012200 (qLw-IA32-1), Gohir.D05G036400 (qUQLw-AZ12-1 and qTNFB-IA27-1), and Gohir.D08G140800 (qLw-AZ9-1), which are mostly associated with fiber length (Supplemental Table 2). We also screened the genes underlying QTL for a compilation of 88 genes mined from the fiber biology literature (see methods). Of these, approximately half (42/88) were found under one or more QTL. Less than 1% of each phenotypic category was composed of genes derived from this list.

#### Plant architecture:

Fourteen QTL were detected for 7 of 10 traits related to plant architecture on 10 chromosomes, 64% of which were from the Arizona population. Nearly half (6) of the fourteen QTL detected relate to stem pubescence, representing four distinct genomic locations and chromosomes; the remaining traits with QTL had only 1-2 QTL each. Particularly notable were the SP QTL located on chromosome A06 (linkage groups IA6 and AZ5), which explained 48.5 and 71.5% of the SP phenotypic variation, respectively. One QTL for plant height (PH) was detected in the D_T_-subgenome (D07; AZ21) in Arizona population, which explained 7.2% of the phenotypic variation (R^2^) and showed additivity. For PH, the TX2094 allele contributes to increasing height, although the two parental alleles work additively (Table 3; Supplemental Table 2).

Homology search of markers associated with these QTL identified 5,646 non-redundant genes in the QTL regions for plant architecture (Supplemental Table 4), with a mean of 433 genes per QTL. For plant height (PH), candidates include (Table 5), among others:a phototropic-responsive NPH3 family protein (Christie *et al.* 2018); a YUC8-like gene (Hentrich *et al.* 2013b); an auxin-responsive family protein (Gallavotti 2013); and tandem duplicates similar to putative far-red impaired responsive (FAR1) family proteins (Tang *et al.* 2013). Approximately 10% of the genes contained within the QTL exhibit differential expression between TX2094 and Maxxa, including a QUASIMODO-like homolog, which leads to a dwarf plant phenotype in Arabidopsis (Orfila *et al.* 2005). Fruiting branch-related traits exhibited 1-2 QTL for branch length (FB1, FB2) and *Plant Height-to-Fruiting Branch Length Ratio* (PHFB1, PHFB2). Interestingly, all QTL for FB1 and PHFB1 were found on D-derived chromosomes, whereas the QTL for FB2 and PHFB2 were found on A-derived chromosomes. Three phototropic-responsive NPH3-like genes are also found within these QTL (Table 5), which have demonstrated roles in *Arabidopsis* phototropism (Christie *et al.* 2018). Also contained within an FB2 QTL is an MKK7-like gene, which is implicated in plant architecture in *Arabidopsis (**Wang and Li 2006**)*, while the single QTL for PHFB1 contains two tandem BIN2-like genes, which can affect plant height in *Arabidopsis* (Li 2005).

**Table 5:** Possible candidates of interest. G. hirsutum gene name and closest Arabidopsis homolog are given (see methods for details). Candidates with amino acid (AA), non-silent SNP (SNP), gene expression (DGE) differences between wild and domesticated cotton are noted in column 5, as are known cotton genes with domestication effects (COTTON) or identified within regions of selective sweeps (SWEEP). Trait categories are listed in columns 6-13, and the traits with QTL that contain that gene are listed

*G. hirsutum gene name*	*Arabidopsis thaliana gene name*	*A. thaliana gene symbol*	*A. thaliana function*	Wild v Dom differences	Plant architecture	Fruiting habit	Phenology	Flower	Seed	Fiber length	Fiber color	Fiber quality
Gohir.A01G101600	AT1G20930	CDKB2;2	cyclin-dependent kinase B2;2		FB2	PHTN,TN,TNFB	FBFF,TNFF		FSW,SW			
Gohir.A01G100800	AT1G79280	AtTPR,NUA	nuclear pore anchor	AA,SNP,SWEEP	FB2	PHTN,TN,TNFB	FBFF,TNFF		FSW,SW			
Gohir.A01G098300	AT5G64330	JK218,NPH3,RPT3	Phototropic-responsive NPH3 amily protein	AA,SNP	FB2	PHTN,TN,TNFB	FBFF,TNFF		FSW,SW			
Gohir.A01G101500	AT1G20980	ATSPL14,FBR6,SPL1R2	squamosa promoter binding protein-like 14	AA,SNP	FB2	PHTN,TN,TNFB	FBFF,TNFF		FSW,SW			
Gohir.A01G143800	AT3G47990	SIS3	SUGAR-INSENSITIVE 3	DGE	FB2	PHTN,TN,TNFB	FBFF,TNFF		FSW,SW			
Gohir.A01G162900	AT1G74110	CYP78A10	cytochrome P450, family 78, subfamily A, polypeptide 10	AA,SNP	FB2	PHTN,TN,TNFB	TNFF		SW			
Gohir.A01G158500	AT1G18350	ATMKK7,BUD1,MKK7	MAP kinase kinase 7		FB2	PHTN,TN,TNFB	TNFF		SW			
Gohir.A01G146200	AT3G19850	none	Phototropic-responsive NPH3 family protein		FB2	PHTN,TN,TNFB	TNFF		SW			
Gohir.A01G154600	AT5G43270	SPL2	squamosa promoter binding protein-like 2		FB2	PHTN,TN,TNFB	TNFF		SW			
Gohir.A11G234300	AT5G19770	TUA3	tubulin alpha-3	AA,DGE,SWEEP	FB2							TrS
Gohir.D07G166500	AT3G07390	AIR12	auxin-responsive family protein		PH	PHTN						
Gohir.D07G165000	AT4G31940	CYP82C4	cytochrome P450, family 82, subfamily C, polypeptide 4	AA,SNP	PH	PHTN						
Gohir.D07G167500	AT3G07500		Far-red impaired responsive (FAR1) family protein		PH	PHTN						
Gohir.D07G167600	AT2G43280		Far-red impaired responsive (FAR1) family protein		PH	PHTN						
Gohir.D07G160100	AT4G28720	YUC8	Flavin-binding monooxygenase family protein		PH	PHTN						
Gohir.D07G164900	AT3G25140	GAUT8,QUA1	Nucleotide-diphospho-sugar transferases superfamily protein	DGE	PH	PHTN						
Gohir.D07G161300	AT5G17580		Phototropic-responsive NPH3 family protein		PH	PHTN						
Gohir.D09G108200	AT1G30440		Phototropic-responsive NPH3 family protein		PHFB1							Fine
Gohir.D09G074400	AT4G18710	ATSK21,BIN2,DWF12,SK21,UCU1	Protein kinase superfamily protein		PHFB1							Fine
Gohir.D09G074500	AT4G18710	ATSK21,BIN2,DWF12,SK21,UCU1	Protein kinase superfamily protein		PHFB1							Fine
Gohir.A12G170400	AT1G13245	DVL4,RTFL17	ROTUNDIFOLIA like 17		SP				FSW			
Gohir.A12G183300	AT5G60970	TCP5	TEOSINTE BRANCHED 1, cycloidea and PCF transcription factor 5		SP				FSW			
Gohir.A06G080100	AT2G26310		Chalcone-flavanone isomerase family protein	AA,SNP	SP					Ln5,Lw,UQLw	Ca,Cb,CL	
Gohir.A06G089400	AT5G47520	AtRABA5a,RABA5a	RAB GTPase homolog A5A	AA	SP					Ln5,Lw,UQLw	Ca,Cb,CL	
Gohir.A06G076500	AT1G77550		tubulin-tyrosine ligases;tubulin-tyrosine ligases	AA,SNP	SP					Ln5,Lw,UQLw	Ca,Cb,CL	
Gohir.A06G111500	AT5G64740	CESA6,E112,IXR2,PRC1	cellulose synthase 6	DGE	SP					Lw	Ca,Cb,CL	
Gohir.A06G108400	AT5G19770	TUA3	tubulin alpha-3		SP					Lw	Ca,Cb,CL	
Gohir.A06G137600	AT1G30040	ATGA2OX2,GA2OX2	gibberellin 2-oxidase	AA,SNP	SP							
Gohir.D06G152200	AT1G11580	ATPMEPCRA,PMEPCRA	methylesterase PCR A	AA,SNP	SP							
Gohir.A13G099200	AT3G13540	ATMYB5,MYB5	myb domain protein 5	AA,SNP,DGE	SP							
Gohir.A06G133200	AT5G45750	AtRABA1c,RABA1c	RAB GTPase homolog A1C		SP							
Gohir.A06G134500	AT5G60860	AtRABA1f,RABA1f	RAB GTPase homolog A1F	AA,SNP	SP							
Gohir.D11G148900	AT5G48460		Actin binding Calponin homology (CH) domain-containing protein	COTTON		NF,TN				L5n		
Gohir.D11G136400	AT2G31200	ADF6,ATADF6	actin depolymerizing factor 6			NF,TN				L5n		
Gohir.D11G119200	AT3G53760	ATGCP4,GCP4	GAMMA-TUBULIN COMPLEX PROTEIN 4			NF,TN				L5n		
Gohir.D07G125100	AT5G07990	CYP75B1,D501,TT7	Cytochrome P450 superfamily protein	DGE		PHTN						
Gohir.D07G187900	AT5G24910	CYP714A1	cytochrome P450, family 714, subfamily A, polypeptide 1	AA,SNP,DGE		PHTN						
Gohir.A01G088300	AT5G25180	CYP71B14	cytochrome P450 family 71 subfamily B polypeptide 14	AA,SNP		PHTN,TN,TNFB			FSW			
Gohir.A01G088800	AT5G25180	CYP71B14	cytochrome P450 family 71 subfamily B polypeptide 14	AA,SNP		PHTN,TN,TNFB			FSW			
Gohir.A01G088500	AT3G26300	CYP71B34	cytochrome P450 family 71 subfamily B polypeptide 34	AA,SNP		PHTN,TN,TNFB			FSW			
Gohir.A01G087900	AT1G13110	CYP71B7	cytochrome P450 family 71 subfamily B polypeptide 7	AA,SNP,DGE		PHTN,TN,TNFB			FSW			
Gohir.A01G091500	AT5G58860	CYP86,CYP86A1	cytochrome P450 family 86 subfamily A polypeptide 1	AA,SNP		PHTN,TN,TNFB			FSW			
Gohir.A01G087100	AT1G50600	SCL5	scarecrow-like 5			PHTN,TN,TNFB			FSW			
Gohir.A01G087000	AT4G26640	AtWRKY20	WRKY family transcription factor family protein			PHTN,TN,TNFB			FSW			
Gohir.A05G289500	AT1G76520		Auxin efflux carrier family protein	DGE		TN		PC				
Gohir.A05G289600	AT1G20925		Auxin efflux carrier family protein	DGE		TN		PC				
Gohir.A05G297200	AT2G39180	ATCRR2,CCR2	CRINKLY4 related 2			TN		PC				
Gohir.A05G291500	AT5G04410	anac078,NAC2	NAC domain containing protein 2			TN		PC				
Gohir.D05G065700	AT4G31590	ATCSLC05,ATCSLC5,CSLC05,CSLC5	Cellulose-synthase-like C5	AA		TNFB				UQLw		
Gohir.D05G028400	AT5G56600	PFN3,PRF3	profilin 3	COTTON		TNFB				UQLw		
Gohir.D05G028500	AT2G19770	PRF5	profilin 5	AA,DGE,COTTON		TNFB				UQLw		
Gohir.D05G093100	AT1G07410	ATRAB-A2B,ATRABA2B,RAB-A2B,RABA2b	RAB GTPase homolog A2B	AA,SNP		TNFB				UQLw		
Gohir.D05G092100	AT5G23860	TUB8	tubulin beta 8	DGE		TNFB				UQLw		
Gohir.D13G132100	AT5G56180	ARP8,ATARP8	actin-related protein 8	AA,SNP			FBFF		FSW	L25n,Ln		Fine
Gohir.D13G121000	AT1G71692	AGL12,XAL1	AGAMOUS-like 12				FBFF		FSW	L25n,Ln		Fine
Gohir.D13G152500	AT5G60860	AtRABA1f,RABA1f	RAB GTPase homolog A1F	AA			FBFF		FSW	L25n,Ln		Fine
Gohir.D13G119200	AT1G71440	PFITFC E	tubulin folding cofactor E / Pfifferling (PFI)				FBFF		FSW	L25n,Ln		Fine
Gohir.D13G156500	AT1G50010	TUA2	tubulin alpha-2 chain				FBFF			L25n,Lw		
Gohir.D13G163700	AT2G21770	CESA09,CESA9	cellulose synthase A9				FBFF,TNFF			L25n,Lw		
Gohir.D13G168700	AT1G55850	ATCSLE1,CSLE1	cellulose synthase like E1	AA,SNP			FBFF,TNFF			L25n,Lw		
Gohir.D13G168800	AT1G55850	ATCSLE1,CSLE1	cellulose synthase like E1	AA,SNP			FBFF,TNFF			L25n,Lw		
Gohir.D13G167800	AT1G50010	TUA2	tubulin alpha-2 chain				FBFF,TNFF			L25n,Lw		
Gohir.D08G056300	AT5G44030	CESA4,IRX5,NWS2	cellulose synthase A4	DGE			GB			L5n,UQLw		
Gohir.D08G063800	AT5G05170	ATCESA3,ATH-B,CESA3,CEV1,IXR1	Cellulose synthase family protein	AA,SNP			GB			L5n,UQLw		
Gohir.D08G063400	AT1G50010	TUA2	tubulin alpha-2 chain				GB			L5n,UQLw		
Gohir.A12G138200	AT4G28250	ATEXPB3,ATHEXP BETA 1.6,EXPB3	expansin B3					CS	FSW			
Gohir.A12G124300	AT1G66350	RGL,RGL1	RGA-like 1					CS	FSW			
Gohir.D12G277100	AT1G10200	WLIM1	GATA type zinc finger transcription factor family protein					CS				
Gohir.D04G119100	AT3G53610	ATRAB8,AtRab8B,AtRABE1a,RAB8	RAB GTPase homolog 8	AA,SNP				PC		L5n		
Gohir.D04G027600	AT5G09810	ACT7	actin 7	AA,SNP				PC		L5n,Ln,Lw		
Gohir.D04G027900	AT5G09810	ACT7	actin 7					PC		L5n,Ln,Lw		
Gohir.D04G031400	AT1G43890	ATRAB-C1,ATRAB18,ATRABC1,RAB18-1	RAB GTPASE HOMOLOG B18	DGE				PC		L5n,Ln,Lw		
Gohir.D04G090100	AT2G37620	AAc1,ACT1	actin 1	AA,DGE				PC		L5n,Lw		
Gohir.D04G108800	AT5G03530	ATRAB,ATRAB ALPHA,ATRAB18B,ATRABC2A	RAB GTPase homolog C2A	SWEEP				PC		L5n,Lw		
Gohir.D04G088800	AT3G57890		Tubulin binding cofactor C domain-containing protein	AA,SNP				PC		L5n,Lw		
Gohir.D04G060300	AT3G55090		ABC-2 type transporter family protein					PC		Ln5,Lw,UQLw		
Gohir.D04G060400	AT3G55090		ABC-2 type transporter family protein	AA,SNP				PC		Ln5,Lw,UQLw		
Gohir.D04G062300	AT5G59890	ADF4,ATADF4	actin depolymerizing factor 4					PC		Ln5,Lw,UQLw		
Gohir.D04G062900	AT3G12110	ACT11	actin-11	AA				PC		Ln5,Lw,UQLw		
Gohir.D04G065100	AT1G07410	ATRAB-A2B,ATRABA2B,RAB-A2B,RABA2b	RAB GTPase homolog A2B	AA,SNP				PC		Ln5,Lw,UQLw		
Gohir.A10G121700	AT5G13930	ATCHS,CHS,TT4	Chalcone and stilbene synthase family protein	DGE				PC				NS
Gohir.A10G121800	AT5G13930	ATCHS,CHS,TT4	Chalcone and stilbene synthase family protein	DGE				PC				NS
Gohir.A05G328100	AT1G05690	BT3	BTB and TAZ domain protein 3	AA				PC,SD				
Gohir.A07G178000	AT3G02350	GAUT9	galacturonosyltransferase 9	SWEEP				PS	FSW,SW	Ln,Lw,UQLw	CL	MR
Gohir.A07G148500	AT2G31200	ADF6,ATADF6	actin depolymerizing factor 6	AA				PS	FSW,SW	Ln,Lw,UQLw		MR
Gohir.A07G157800	AT3G07330	ATCSLC06,ATCSLC6,CSLC06,CSLC6	Cellulose-synthase-like C6	AA,SNP,DGE				PS	FSW,SW	Ln,Lw,UQLw		MR
Gohir.A07G137700	AT3G61760	ADL1B,DL1B	DYNAMIN-like 1B	AA,SNP				PS	FSW,SW	Ln,Lw,UQLw		MR
Gohir.A07G127600	AT2G47460	ATMYB12,MYB12,PFG1	myb domain protein 12	AA				PS	FSW,SW	Ln,Lw,UQLw		MR
Gohir.A07G146700	AT2G45190	AFO,FIL,YAB1	Plant-specific transcription factor YABBY family protein	AA,SNP,SWEEP				PS	FSW,SW	Ln,Lw,UQLw		MR
Gohir.A07G135700	AT1G01200	ATRAB-A3,ATRABA3,RABA3	RAB GTPase homolog A3					PS	FSW,SW	Ln,Lw,UQLw		MR
Gohir.A07G159800	AT3G07410	AtRABA5b,RABA5b	RAB GTPase homolog A5B					PS	FSW,SW	Ln,Lw,UQLw		MR
Gohir.A07G162600	AT4G17170	AT-RAB2,ATRAB-B1B,ATRAB2A,ATRABB1C	RAB GTPase homolog B1C					PS	FSW,SW	Ln,Lw,UQLw		MR
Gohir.A07G118300	AT5G12250	TUB6	beta-6 tubulin					PS	SW	Lw,UQLw		
Gohir.A07G118400	AT2G29550	TUB7	tubulin beta-7 chain					PS	SW	Lw,UQLw		
Gohir.A04G056700	AT2G37620	AAc1,ACT1	actin 1					SD		Lw		
Gohir.A04G058700	AT3G57890		Tubulin binding cofactor C domain-containing protein					SD		Lw		
Gohir.A08G182500	AT5G12250	TUB6	beta-6 tubulin	DGE,COTTON				SD			Ca,Cb,CL	
Gohir.A08G144300	AT5G05170	ATCESA3,ATH-B,CESA3,CEV1,IXR1	Cellulose synthase family protein	DGE				SD			Ca,Cb,CL	
Gohir.A08G137800	AT1G02050	LAP6	Chalcone and stilbene synthase family protein	AA,SNP				SD			Ca,Cb,CL	
Gohir.A08G186100	AT3G63170		Chalcone-flavanone isomerase family protein	AA,SNP				SD			Ca,Cb,CL	
Gohir.A08G192500	AT4G28720	YUC8	Flavin-binding monooxygenase family protein					SD			Ca,Cb,CL	
Gohir.D10G150700	AT4G24000	ATCSLG2,CSLG2	cellulose synthase like G2	AA,SNP,SWEEP					AL	L5n,LnCV		
Gohir.D05G156700	AT4G18780	ATCESA8,CESA8,IRX1,LEW2	cellulose synthase family protein	AA,SNP,DGE					AL	UQLw		
Gohir.D05G134800	AT5G42080	ADL1,ADL1A,AG68,DL1,DRP1A,RSW9	dynamin-like protein						AL	UQLw		
Gohir.D05G156200	AT5G45750	AtRABA1c,RABA1c	RAB GTPase homolog A1C	AA					AL	UQLw		
Gohir.D05G111300	AT5G23860	TUB8	tubulin beta 8	AA					AL	UQLw		
Gohir.D05G210400	AT1G77980	AGL66	AGAMOUS-like 66	AA,SNP					AL			TrS
Gohir.D10G130800	AT1G05810	ARA,ARA-1,ATRAB11D,ATRABA5E	RAB GTPase homolog A5E	AA,SNP					AL,FSW	L5n,LnCV		
Gohir.D13G092900	AT3G46060	ARA-3,ARA3,ATRAB8A,ATRABE1C	RAB GTPase homolog 8A						FSW	L25n,Ln		
Gohir.D13G102800	AT5G19770	TUA3	tubulin alpha-3						FSW	L25n,Ln		
Gohir.D13G103900	AT3G10220		tubulin folding cofactor B	AA					FSW	L25n,Ln		
Gohir.D10G111800	AT1G13180	ARP3,ATARP3,DIS1	Actin-like ATPase superfamily protein						FSW	L5n,LnCV		SFCn
Gohir.D10G111500	AT2G30910	ARPC1,ARPC1A	actin-related protein C1A						FSW	L5n,LnCV		SFCn
Gohir.D10G109100	AT1G43890	ATRAB-C1,ATRAB18,RAB18-1	RAB GTPASE HOMOLOG B18						FSW	L5n,LnCV		SFCn
Gohir.A07G190000	AT5G42080	ADL1,ADL1A,AG68,DL1,DRP1A,RSW9	dynamin-like protein						FSW	Ln,Lw,UQLw	CL	MR
Gohir.A07G189000	AT1G12780	ATUGE1,UGE1	UDP-D-glucose/UDP-D-galactose 4-epimerase 1	AA,SNP					FSW	Ln,Lw,UQLw	CL	MR
Gohir.A07G192300	AT4G12730	FLA2	FASCICLIN-like arabinogalactan 2						FSW	Lw,UQLw	CL	MR
Gohir.A07G194000	AT4G17170	AT-RAB2,ATRAB-B1B,ATRAB2A,ATRABB1C	RAB GTPase homolog B1C						FSW	Lw,UQLw	CL	MR
Gohir.A07G193900	AT2G04160	AIR3	Subtilisin-like serine endopeptidase family protein	AA					FSW	Lw,UQLw	CL	MR
Gohir.A05G153500	AT5G45750	AtRABA1c,RABA1c	RAB GTPase homolog A1C	AA					FSW,SCW	UQLw		
Gohir.D08G120800	AT5G09810	ACT7	actin 7							L5n		
Gohir.D08G100500	AT1G60430	ARPC3	actin-related protein C3							L5n		
Gohir.D11G245500	AT1G55850	ATCSLE1,CSLE1	cellulose synthase like E1							L5n		
Gohir.D11G245600	AT1G55850	ATCSLE1,CSLE1	cellulose synthase like E1							L5n		
Gohir.D11G245700	AT1G55850	ATCSLE1,CSLE1	cellulose synthase like E1	AA,SNP,DGE						L5n		
Gohir.D11G245800	AT1G55850	ATCSLE1,CSLE1	cellulose synthase like E1							L5n		
Gohir.D11G245900	AT1G55850	ATCSLE1,CSLE1	cellulose synthase like E1							L5n		
Gohir.D11G161300	AT2G32540	ATCSLB04,ATCSLB4,CSLB04	cellulose synthase-like B4	AA,SNP						L5n		
Gohir.D08G086000	AT3G53760	ATGCP4,GCP4	GAMMA-TUBULIN COMPLEX PROTEIN 4							L5n		
Gohir.D08G105500	AT1G50010	TUA2	tubulin alpha-2 chain							L5n		
Gohir.D11G245300	AT5G19770	TUA3	tubulin alpha-3							L5n		
Gohir.D08G105000	AT5G62690	TUB2	tubulin beta chain 2	DGE						L5n		
Gohir.D11G253600	AT5G62690	TUB2	tubulin beta chain 2							L5n		
Gohir.D08G242000	AT3G03050	ATCSLD3,CSLD3,KJK	cellulose synthase-like D3							Ln,Lw		
Gohir.D01G125700	AT2G37620	AAc1,ACT1	actin 1	SWEEP						Ln25,Ln5,Lw,UQLw		
Gohir.D01G166800	AT5G09810	ACT7	actin 7	AA						Ln25,Ln5,Lw,UQLw		
Gohir.D01G157800	AT2G16700	ADF5,ATADF5	actin depolymerizing factor 5							Ln25,Ln5,Lw,UQLw		
Gohir.D01G139500	AT1G14830	ADL1C,ADL5,DL1C,DRP1C	DYNAMIN-like 1C	AA,SNP						Ln25,Ln5,Lw,UQLw		
Gohir.D01G126500	AT3G12160	ATRABA4D,RABA4D	RAB GTPase homolog A4D	SWEEP						Ln25,Ln5,Lw,UQLw		
Gohir.D01G129900	AT5G03530	ATRAB,ATRAB ALPHA,ATRAB18B,ATRABC2A	RAB GTPase homolog C2A							Ln25,Ln5,Lw,UQLw		
Gohir.D01G126200	AT3G57890		Tubulin binding cofactor C domain-containing protein	AA,SNP,SWEEP						Ln25,Ln5,Lw,UQLw		
Gohir.D01G184700	AT3G57890		Tubulin binding cofactor C domain-containing protein	AA						Ln25,Ln5,Lw,UQLw		
Gohir.D01G196200	AT2G36250	ATFTSZ2-1,FTSZ2-1	Tubulin/FtsZ family protein							Ln25,Ln5,Lw,UQLw		
Gohir.A06G062700	AT4G13260	YUC2	Flavin-binding monooxygenase family protein	AA						Ln5,Lw,UQLw	Ca,Cb,CL	
Gohir.A06G068300	AT2G19760	PFN1,PRF1	profilin 1	COTTON						Ln5,Lw,UQLw	Ca,Cb,CL	
Gohir.A06G068400	AT4G29340	PRF4	profilin 4	COTTON						Ln5,Lw,UQLw	Ca,Cb,CL	
Gohir.D11G231100	AT5G09810	ACT7	actin 7							Ln5,Lw,UQLw		
Gohir.D11G226600	AT5G64740	CESA6,E112,IXR2,PRC1	cellulose synthase 6							Ln5,Lw,UQLw		
Gohir.D11G219500	AT5G65270	AtRABA4a,RABA4a	RAB GTPase homolog A4A							Ln5,Lw,UQLw		
Gohir.D11G221500	AT5G10260	AtRABH1e,RABH1e	RAB GTPase homolog H1E	SWEEP						Ln5,Lw,UQLw		
Gohir.D12G155800	AT1G14830	ADL1C,ADL5,DL1C,DRP1C	DYNAMIN-like 1C							LnCV	CL	
Gohir.D08G199700	AT4G00680	ADF8	actin depolymerizing factor 8							Lw		
Gohir.D08G165000	AT3G60830	ARP7,ATARP7	actin-related protein 7							Lw		
Gohir.D08G201000	AT5G12250	TUB6	beta-6 tubulin	DGE						Lw		
Gohir.D08G165300	AT5G05170	ATCESA3,ATH-B,CESA3,CEV1,IXR1	Cellulose synthase family protein	DGE						Lw		
Gohir.D08G125700	AT5G42080	ADL1,ADL1A,AG68,DL1,DRP1A,RSW9	dynamin-like protein	AA						Lw		
Gohir.D08G169100	AT4G19400		Profilin family protein	AA, COTTON						Lw		
Gohir.A04G037000	AT5G60860	AtRABA1f,RABA1f	RAB GTPase homolog A1F							Lw		
Gohir.D08G166800	AT5G47960	ATRABA4C,RABA4C,SMG1	RAB GTPase homolog A4C							Lw		
Gohir.D08G199800	AT5G23860	TUB8	tubulin beta 8	AA						Lw		
Gohir.D09G042600	AT5G23860	TUB8	tubulin beta 8	AA,SNP								Fine
Gohir.A07G205900	AT3G29030	ATEXP5,ATEXPA5,ATHEXP ALPHA 1.4	expansin A5									MR
Gohir.A07G209500	AT1G06780	GAUT6	galacturonosyltransferase 6	AA,DGE								MR

Stem pubescence had both the highest number of QTL and candidate genes, many of which have predicted functions in trichome and/or cell wall development, as well as amino acid changes between TX2094 and Acala Maxxa. One candidate is a predicted Myb 5-like gene (Table 5), which functions in trichome development in *Arabidopsis*. Two other candidates include two RAB GTPase-like genes, a gibberellin 2-oxidase-like gene, and a methylesterase-like gene, all of which have amino acid changes; genes involved in these processes are associated with cell wall metabolism or related pathways in *Arabidopsis* (Lycett 2008; Bischoff *et al.* 2010) and cotton (Xiao *et al.* 2019). Although somewhat further from the QTL peak, a cellulose synthase 6-like gene was found within the SP QTL, which is relevant to trichome development (Haigler *et al.* 2009; Betancur *et al.* 2010; Nixon *et al.* 2016).

#### Fruiting habit and Phenology:

Nineteen QTL were detected for seven traits related to fruiting habit (4 traits) and phenology (3 traits; see Table 1), split evenly between subgenomes and scattered across 10 chromosomes. Five and three Fruiting Habit QTL were identified for Total Number of Nodes (TN) and Plant Height-to-Total Number of Nodes Ratio (PH_by_TN), respectively, in the Iowa and Arizona populations (Supplemental Table 2). Most QTL for PH_by_TN showed additivity, whereas only one exhibited additivity for TN; the remaining four QTL exhibited partial- or over-dominance. Three QTL were detected for Total Number of Non-Fruiting Branches (TNFB) dispersed across three chromosomes (2 A_T_ and 1 D_T_) and occurring in both subpopulations (2 Iowa, 1 Arizona), whereas a single QTL was found for Total Number of Nodes to First Fruiting Branch (NF) in the Arizona subpopulation, which was found on chromosome D11 and explained 35% of the variation for the trait.

Two phenology QTL were identified for Total Number of Nodes at First Flower (TNFF) in the Iowa population only. The two QTL for TNFF were either partial or over-dominance and explained ∼7% of the phenotypic variation each, whereas the three QTL for FBFF were either dominant, overdominant, or additive, explaining between 7.9–14.9% of the variation. Interestingly, while the final Phenology trait, Total Number of Green Bolls Retained after 30 days + 4 week interval (GB) exhibited two QTL (Arizona subpopulation only), one from each subgenome, the chromosomes were not homeologous (*i.e.*, were not homologous in the diploid progenitors).

Homology searches of QTL-associated markers recovered 5,136 non-redundant genes in the QTL intervals controlling fruiting habit and 2,661 genes in the intervals controlling phenology. Although many of the same chromosomes were implicated in both trait categories, only 714 genes are shared between the two. Nearly half of the genes recovered for both traits exhibited SNPs with potential effects (*e.g.*, amino acid changes) between TX2094 and Acala Maxxa (45% and 49% for Fruiting Habit and Phenology, respectively); however, few genes exhibited differential expression (8% in each; Supplemental Table 4). Putative candidates for PH_by_TN include two genes similar to *Arabidopsis* WRKY and GRAS transcription factors (Table 5) and at least nine cytochrome P450-like genes, which are part of a relatively large superfamily of genes with diverse metabolic roles (Mizutani and Ohta 2010; Mizutani 2012); most of these cytochrome P450-like genes (6) have predicted amino acid changes between TX2094 and Acala Maxxa.Total number of nodes (TN) QTL candidate genes include two differentially expressed auxin efflux carrier family proteins; a differentially expressed SIS3-like homolog; and a CCR-related gene (Table 5). Homologs of SIS3 are involved in the growth response to high concentrations of exogenous sugars (Huang *et al.* 2010)members of the CCR gene family may be involved in lignin biosynthesis during development (Lauvergeat *et al.* 2001). Several genes are found associated with the TN QTL in regions that overlap the TNFB QTL, including a homolog of SPL2, which is involved in shoot maturation and the transition to flowering (Shikata *et al.* 2009); a nuclear pore anchor, whose *Arabidopsis* homolog affects flowering time regulation and other developmental processes (Xu *et al.* 2007); and two adjacent genes, a squamosa promoter binding protein-like and a cyclin-dependent kinase B2;2-like gene,, both of which are involved in plant growth and development (Andersen *et al.* 2008; Jorgensen and Preston 2014). For the single QTL involved in NF, no obvious candidate genes were noted; however, 46% of the 660 genes in the QTL regions were affected by non-conservative SNPs (see methods), including 29% with amino acid changes. Interestingly, many Fruiting habit QTL candidates overlap those found in Plant architecture (Table 5), which may reflect an overlap in developmental programs.

While three traits representing the Phenology trait category each recovered QTL (*i.e.*, FBFF, GB, and TNFF), the QTL for FBFF and TNFF largely overlapped. Most QTL regions encompassed by TNFF were also found for FBFF, except for part of chromosome A01, where the FBFF QTL is more narrowly predicted than in TNFF. This region of chromosome A01 also has many overlapping QTL for Fruiting habit and other Phenology traits (*i.e.*, PHTN, TN, TNFB), which may indicate that it is a notable region for plant growth and development. The other QTL for FBFF were located solely on the D_T_ chromosomes, and includes an AGAMOUS-like gene (Table 5), which could act responsively to plant hormones and have function in regulating fruit formation in cotton (de Moura *et al.* 2017). Interestingly, the QTL for FBFF on chromosome D13 overlaps with QTL for Fiber Length and therefore contains some fiber-relevant genes (Table 5), including a tubulin-related gene . Similarly, one of the two QTL for GB entirely overlaps with 1-2 Fiber length QTL on chromosome D08, while the other QTL completely overlaps with the Plant Architecture QTL PHFB2 (see above). These overlapping QTL regions may also reflect overlap in developmental programs between fiber development, plant architecture and growth, and fruit retention.

#### Flower:

Seventeen QTL were identified for four floral traits, which individually explain 4.6–66.1% of the phenotypic variation and most of which exhibited varying degrees of dominance. Four QTL were detected for Average Stigma Distance (SD), two from each population, on four different chromosomes (A04, A05, A08 and D11). Four QTL were also identified for Curly Style (CS) from the Iowa population only, with the curly allele typically originating from TX2094. Seven QTL were detected for Pollen Color (PC) on two A and two D chromosomes (A05, A10, D04, and D05); presence of TX2094 alleles generated more yellow pollen (Supplemental Table S2). Finally, two QTL were detected for the presence of a petal spot (PS; chromosome A07), a TX2094-derived trait.

Candidate gene searches revealed 8,272 genes in the QTL intervals for floral traits. The QTL for curly style exhibited several genes related to cell wall formation and/or organization, which may be involved in conferring the curly phenotype (Table 5). These include an RGA-like gene that may play a role in regulating organ development (Wang *et al.* 2009); an expansin B3-like gene which may be involved in cell wall expansion mediation (Shcherban *et al.* 1995; Lee *et al.* 2001); and a WLIM1-like transcription factor whose *Arabidopsis* homolog regulates cytoskeletal organization via interaction with actin filaments (Papuga *et al.* 2010). Likewise, several notable genes were detected for pollen color. Two of these are arrayed in tandem and are putative ABC-2 type transporter-like genes; this gene family participates in pollen wall synthesis, as observed in *Arabidopsis* (Yadav *et al.* 2014). A second tandem array of two putative homologs of chalcone synthase was also found for PC, with both members exhibiting differential expression between Acala Maxxa and TX2094 (albeit measured in fiber only). An additional PC-related gene is an NAC-like gene with a possible role in regulating flavonoid biosynthesis (Morishita *et al.* 2009). Similarly, the single notable gene within the QTL for PS is a myb domain protein whose *Arabidopsis* homolog is involved in flavonoid biosynthesis (Wang *et al.* 2016b). The QTL for average stigma distance includes a single gene of interest, a transcription factor which plays a role in male and female gametophyte development (Robert *et al.* 2009).

#### Seed:

Sixteen QTL were identified representing five of the seven seed-related traits (Supplemental Table 2), which individually explain 5.6–12.87% of the variance per trait. The trait 50 Fuzzy Seed Weight (FSW) had the most QTL (7), distributed over 6 chromosomes. The remaining traits had 1-3 associated QTL, most having a positive effect allele from the domesticated Acala Maxxa parent. Most seed QTL reside on A_T_ subgenome chromosomes (10 out of 16, including 5 of the QTL for FSW).

QTL for Seed-related traits contain 9,116 candidate genes. For the fuzzy seed weight QTL regions, these include a UDP-D-glucose/-galactose 4-epimerase and several FASCICLIN-like arabinogalactans (FLA), including a FLA2-like gene (Table 5). Both of these exhibit up-regulation in domesticated (*vs.* wild) cottons (Yoo and Wendel 2014) and have *Arabidopsis* homologs that function in cell wall biosynthesis. Also included in the QTL region is a Pfifferling (PFI)-like homolog, which functions in seed (embryo) development in *Arabidopsis* (Steinborn *et al.* 2002), and an expansion (EXPA5)-like homolog, which may act to mediate cell wall expansion (Shcherban *et al.* 1995; Lee *et al.* 2001). Notably, these genes all belong to the FSW QTL, which overlaps in these regions with QTL for fiber traits. An additional two candidate genes within the FSW QTL have possible roles in fruit formation: a DVL-homolog that may confer phenotypic changes in fruit and inflorescence (Wen *et al.* 2004), and an AGAMOUS 12-like gene whose family has a suggested role in cotton fruit formation (de Moura *et al.* 2017). The only other notable candidate gene within the Seed QTL is another AGAMOUS-like gene, which was found within the QTL for AL.

#### Fiber length:

Fiber-related characteristics were among the obvious phenotypic targets during domestication of cotton. Not surprisingly, therefore, 54 QTL were detected for fiber-related traits (*i.e.*, length, color, and measures of quality), of which 33 (61%) were for fiber length (Supplemental Table 2). As observed in some other populations, a majority of these (76% or 25 QTL) were located in the subgenome (D_T_) derived from the parental diploid that has short, unspinnable fiber. These QTL were dispersed over 9 of the 13 D_T_ chromosomes and 4 of the 13 A_T_-derived chromosomes, individually explaining from 7.2 to 17.5% of the phenotypic variation. Despite having far fewer QTL, the A_T_-subgenome exhibited QTL for four of the seven length traits evaluated (Supplemental Table 2). Only 4 of the A_T_-subgenome QTL explained more than 10% of the variation (*vs.* 12 D_T_ QTL) and only one was in the top 5 fiber-length related QTL, explaining at most 12.1% of the trait variation. Conversely, nearly half of the QTL found on D_T_-subgenome chromosomes (Supplemental Table 2) individually explain over 10% of the phenotypic variation (R^2^) for their categories (12 out of 25 D_T_ QTL).

Candidate gene searches for fiber length QTL revealed several possibilities (Table 5), including 19 cellulose synthase-like genes, most of which (17) are found on the D_T_ chromosomes and five of which clustered on chromosome D11. The middle gene in this cluster, Gohir.D11G245700, exhibited both amino acid changes and differential gene expression between wild and domesticated *G. hirsutum*, supporting a possible role in fiber domestication. Differential expression was also found for four other cellulose synthase-like genes, including both genes found on the A_T_ chromosomes . Because many of the fiber QTL overlap, nearly half (8) of the cellulose synthase genes were associated with multiple Fiber length QTL (mean = 1.5 QTL). Interestingly, an additional cellulose synthase-like gene (Gohir.A08G144300) was also differentially expressed between wild and domesticated cotton; however, this gene was not contained within any fiber length QTL, but was rather found associated with multiple fiber color QTL and one for Average Stigma Distance (Supplemental Table 4). Similarly, several genes typically associated with flavonoid production (*e.g.*, chalcone-flavanone isomerase) were found within the fiber length QTL rather than the QTL for fiber color where they would be expected to influence the brown coloration found in wild fibers.

As expected, many additional candidate genes involved in cytoskeleton/cell wall formation or trichome development were found, including several genes with known associations with fiber development (Table 5). Twenty-five tubulin related genes were found associated with fiber length QTL, including eight beta tubulin-like genes. Beta tubulin genes are relevant to cell wall development because they orient the cellulose microfibrils (Spokevicius *et al.* 2007), a major component of secondary cell walls. Three of the beta tubulin-like genes exhibit differential expression between wild and domesticated cotton fiber, and each is associated with a different QTL trait (Table 5). Eighteen actin-related genes were also found within the fiber QTL, including one with a known role in fiber elongation and secondary wall synthesis (Gohir.D11G148900; (Zhang *et al.* 2017)); however, no differential expression or SNPs with predicted functional consequences were detected between wild and domesticated cotton for this gene. Five profilin homologs were associated with fiber length; profilin expression has previously been associated with fiber domestication (Bao *et al.* 2011). Six dynamin(DL1)-like proteins were also associated with Fiber length, along with 22 RAB GTPase-like genes (Table 5). In *Arabidopsis*, these genes influence cell wall composition (both) and cellular expansion (DL1) (Collings *et al.* 2008). Notably, the DL1-like candidate and one RAB GTPase-like candidate exhibits differential expression between wild and domesticated cotton fiber. Finally, a YABBY1 transcription factor-like gene was associated with fiber length whose *Arabidopsis* homeolog is exclusively expressed in trichomes (Schliep *et al.* 2010). This candidate gene also exhibits an amino acid change between wild and domesticated cotton.

#### Fiber color:

Fiber color is conferred by the accumulation of flavonoids in mature fibers (Hua *et al.* 2007; Xiao *et al.* 2007, 2014; Li *et al.* 2012a; Feng *et al.* 2013; Tuttle *et al.* 2015). Thirteen QTL were detected for the three fiber color traits evaluated: mean L* (bright/dark), mean a* (green/red), and mean b* (blue/yellow). Many of these on chromosomes A06 and A08 overlapped between populations and traits, and therefore aggregate into two distinct QTL hotspots. The QTL on chromosome A06 were typically of major effect, individually explaining from 43.8 to 79.9% of the phenotypic variation, whereas those on chromosome A08 typically explained less than 10% of the variation (from 5.1 to 12.9%; mean 8.8%). Two flavin-binding monooxygenase family (YUCCA)-like proteins were found within the color QTL detected here, one each on chromosomes A06 and A08 (Table 5). *Arabidopsis* homologs of the YUCCA family function in the production of auxin (Hentrich *et al.* 2013a, 2013b), a key regulator of plant development that may also be involved in the regulation of flavonol synthesis (Lewis *et al.* 2011). Likewise, a chalcone-flavanone isomerase family-like protein was found within the color QTL on both A06 and A08, which also functions in flavonoid biosynthesis in *Arabidopsis* (Jiang *et al.* 2015). Chromosome A08 has an additional flavonol-related candidate gene, *i.e.*, a chalcone and stilbene synthase family protein. Interestingly, while chromosomes A06 and A08 have loci with predicted relevance to fiber color, the QTL on chromosomes A07, D07, and D12 do not exhibit any notable candidates; however, the color QTL for chromosomes A07 and D12 do overlap QTL for fiber length and fiber quality in which there exist several genes that may influence fiber morphology (Table 5). These include the previously mentioned dynamin-like gene, a gene similar to FASCICLIN-like arabinogalactan that has been implicated in fiber domestication (Yoo and Wendel 2014) and cell wall biosynthesis (MacMillan *et al.* 2010), and a TUB6-like gene. Whether the overlap of these QTL is coincidence or suggests an overlap in the genetic networks conferring different fiber traits is unknown and will require future research on the fiber development network.

#### Other fiber qualities:

While a total of 14 “other” measures of fiber quality were evaluated (Table 1), only five traits produced QTL (8 QTL), namely, Fineness, Maturity Ratio, Nep Size, Short Fiber Content by Number, and Trash Size. Each trait was associated with 1-2 QTL each for a total of 8 QTL located on as many chromosomes. Several candidates affecting cell wall composition and synthesis were found within these two regions (Table 5). These include two tubulin-like genes, Gohir.A11G234300 and Gohir.D09G042600, which exhibit differential expression and amino acid changes, respectively. An actin-like ATPase found in this region is similar to the *Arabidopsis* ARP3 gene, which controls trichome shape (Mathur *et al.* 2003). The region also includes a subtilisin protease-like candidate; subtilisin proteases have been associated with cell wall composition in *Arabidopsis thaliana*, specifically the mucilage content of cell walls (Rautengarten *et al.* 2008). Two additional candidates are galacturonosyltransferase (GAUT)-like genes (Table 5), whose *Arabidopsis thaliana* homologs influence cell wall composition by controlling pectin biosynthesis (Caffall 2008; Caffall *et al.* 2009; Atmodjo *et al.* 2011).

### Comparison of putative QTL between subpopulations, between subgenomes, and among chromosomes

The F_2_ seed derived from a single cross between *G. hirsutum* accessions TX2094 and Acala Maxxa were planted in two different greenhouse environments, in Maricopa, AZ and Ames, IA (see methods). The 120 total QTL detected were nearly evenly divided between the two subpopulations, with Arizona recovering slightly more QTL (67 QTL, or 56%) than Iowa. While the number of QTL recovered in each subpopulation was similar, only 22 QTL were declared as coincident QTL between the two locations, and eight of them shared peak markers. Likewise, while both populations detected QTL on a similar number of chromosomes (20 and 21 in Arizona and Iowa, respectively), approximately 30% of chromosomes (7) had QTL from only one population. On average, the QTL detected in Iowa had a slightly more narrow range (Supplemental Table 2), both overall (13.2 *vs.* 19.1 cM, or 14 *vs.* 39 Mb) and when only considering QTL regions with the same peak marker (18.6 *vs.* 20.7 cM, or 5 *vs.* 30 Mb). Slight and opposing subgenome biases were found for the chromosomes recovered from each subpopulation, with Iowa recovering QTL on 11 A_T_ and 10 D_T_ chromosomes, whereas Arizona recovered QTL on 9 A_T_ and 11 D_T_ chromosomes.

The QTL peaks shared between the Iowa and Arizona subpopulations were exclusively associated with fiber color (2 peak markers, 4 QTL regions; Supplemental Table 2), with the remaining seven coincident regions influencing fiber length (1 shared QTL region), flower (3 shared QTL regions), seed (1 shared QTL region), and plant architecture (2 shared QTL regions). Eight of the 11 coincident QTL regions were located on A_T_-derived chromosomes, with chromosome A06 represented most frequently (3 shared QTL regions; Figure 2). Three of the 8 trait categories surveyed had no shared QTL regions, *i.e.*, Fiber Quality, Fruiting Habit, and Phenology; this is possibly due in part to these being the categories with the fewest QTL reported (Supplemental Table 2).

The distribution and total length of the 120 QTL was nearly equivalent between the two polyploid subgenomes (59A:61D); however, when QTL redundancy between subpopulations is considered, this proportion becomes slightly D-biased (51A:58D). This may be due to the bias toward A_T_ chromosomes in shared QTL and a slight overrepresentation of D_T_-derived QTL in the Arizona population (32A:35D). Both the mean and median length of A_T_ derived QTL are larger than for D_T_ derived QTL (36.5 *vs.* 16 Mb, respectively, for mean, and 31 *vs.* 8 Mb for median), which is likely a consequence of the larger genome size (twofold) inherited from the A diploid parent. Slightly more than half of the categories (*i.e.*, fiber color, flower, fruiting habit, and seed) had more A_T_ QTL, with fiber color exhibiting the largest bias (85% A_T_-derived QTL). Fiber length exhibited the next greatest bias, albeit for the opposite subgenome; *i.e.*, approximately 76% (25) of fiber length QTL are D_T_-derived. In fact, approximately half of the total D_T_-derived QTL are associated with fiber length (∼41% overall). Interestingly, because the fiber quality category also contained more D_T_-derived QTL (3A:5D), these two fiber categories together accounting for nearly half of the QTL from D_T_ subgenome chromosomes and over 73% of the QTL for these categories. This observation is congruent with some previous research that has suggested D-genome recruitment during fiber domestication.

## Discussion

### QTL lability and the complex genetic architecture of cotton domestication phenotypes

The molecular underpinnings of the domesticated cotton fiber phenotype are of substantial interest from both evolutionary and economic standpoints. Because a cotton “fiber” is a highly exaggerated single-celled structure, it provides a unique model for the evolutionary and developmental transformations that are possible in a single cell. Economically, cotton fibers are central to a multi-billion dollar and globally vital industry, one that has a vested interest in manipulating the genetics of domesticated fiber. Consequently, myriad studies have attempted to reveal the key players in fiber development. The results of these experiments and analyses have been diverse and often in conflict, underscoring the complex nature of cotton fiber biology and also the diverse suite of populations that have variously been employed. Comparison between the present research and previously generated QTL suffers from this same complexity. Many of the phenotypic traits evaluated here have been evaluated in other crosses and under different conditions, as summarized in the Cotton QTL Database v. 2.3 (Said *et al.* 2015a) and CottonGen (Yu *et al.* 2014). As noted by others, QTL results of an individual study (such as the one presented here) are frequently incongruent with QTL results from other crosses grown under different conditions (Rong *et al.* 2007; Lacape *et al.* 2010; Said *et al.* 2015b, 2015a). This observation is clear from our results alone, where less than half of the QTL were shared across two similar environments. When extended to previous QTL results, even our most robust QTL (*i.e.*, fiber color, chromosome A06) exhibit more complicated inheritance; *i.e.*, the Cotton QTL Database lists 62 QTL for fiber color spread across 21 of the 26 cotton chromosomes whereas we detect a single chromosome of major effect and only 4 of lesser effect for both environments. A notable difference between ours and previous studies, however, is that ours was designed to capture the array of changes that characterize the transformation of the truly wild form of *G. hirsutum* into the modern elite cultivars that presently comprise the modern annualized crop plant. This cross should capture the major differences between wild and domesticated forms of *G. hirsutum*, whereas previous research has focused on differences between either (1) elite lines of the independently domesticated species *G. hirsutum* and *G. barbadense* (*i.e.*, Pima cotton), or (2) between *G. hirsutum* landraces and/or elite cultivars, which reflect differences in improvement rather than those accompanying initial domestication.

Notwithstanding these substantive differences among studies, both the results presented here and earlier indicate that the genetic architecture underlying fiber morphology and development (among other domestication phenotypes) is complex and is responsive to environmental conditions. Consequently, uncovering QTL represent an important yet insufficient step in disentangling the genetic underpinnings of fiber development and cotton domestication. The complex interactions among genes important to understanding the QTL recovered remain to be elucidated, but many important enabling tools for such analyses have been developed. For example, gene coexpression network analyses can reveal modules of interconnected genes involved in key traits, as shown for cottonseed (Hu *et al.* 2016) and fiber (Joseph P. Gallagher, Corrinne E. Grover, Guanjing Hu, Josef J. Jareczek, Jonathan F. Wendel, unpublished data), using the comparative context of wild *vs.* domesticated *G. hirsutum*. In these examples, domestication appears to have increased the coordinated expression among genes and gene modules relevant to domesticated phenotypes. Research on *cis*/*trans* regulatory differences between wild and domesticated *G. hirsutum* (Bao, Hu, *et al.* 2019) indicates that changes in both *cis* and *trans* regulation have occurred during domestication, which are significantly enriched with fiber QTL genes reported here. Notably, regulatory variations are frequently associated with environmental responsiveness (Cubillos *et al.* 2014; Lovell *et al.* 2016; Waters *et al.* 2017) and therefore may underlie the environmental variability of QTL as reported.

### Multiple sources of information can narrow candidate gene identification

A primary goal of QTL analyses is to uncover the genomic basis of phenotypic differences. In many cases, QTL regions encompass a large region of the genome, and hence contain many genes. Here, each individual QTL recovered between 14 and 1,678 genes (mean = 531), resulting in 1,782 - 11,807 possible candidate genes for each phenotype (Supplemental Table 2). In the present analysis, we narrow the candidate genes to focus on those genes with secondary evidence, *i.e.*, DGE, amino acid changes, transcription factors, and/or those with relevant functions in related species. The genes mentioned here as candidates, while not exhaustive, represent possible causative sources for their respective phenotypes. The strength of these candidates, however, is limited by the information available. For the fiber QTL, we were able to leverage existing expression information for the accessions used in the QTL mapping cross, which provides additional evidence supporting individual genes as candidates. A caveat, however, is that since the expression sampling was completed for an independent project and QTL are often environmentally labile, genes exhibiting differential expression (or lack thereof) in the dataset used here may not represent the expression patterns that would be observed in the individuals used in the initial QTL cross and grown under the conditions of the QTL subpopulations. Furthermore, differential expression data were only available for two timepoints during fiber development, albeit key timepoints (Haigler *et al.* 2012). Future QTL research may be improved by integrating multiple data types from the outset, including expression from tissues relevant to the phenotypes evaluated for each parent grown in each environment; however, the results of the present were improved (for the fiber phenotype) by considering the data available.

### Implications for domestication and future prospects

Domestication is a complex process involving a multiplicity of traits and the coordinated alteration of gene expression for numerous genes, for all but the simplest of traits (Olsen and Wendel 2013a, 2013b; Meyer and Purugganan 2013; Kantar *et al.* 2017; Purugganan 2019). With respect to cotton, a large number of QTL analyses have been conducted, specifically focused on economically valuable fiber characteristics, with some interest in other agronomically important phenotypes. These analyses have used either different species (Jiang *et al.* 1998; Paterson *et al.* 2003; Mei *et al.* 2004; Lacape *et al.* 2005, 2010; Chee *et al.* 2005a, 2005b; Draye *et al.* 2005; Rong *et al.* 2007; Said *et al.* 2015b, 2015a; Wang *et al.* 2016a, 2017a, 2017c) or different cultivated lines of the same species (Ulloa *et al.* 2005; Zhang *et al.* 2005; Shen *et al.* 2006; Qin *et al.* 2008; Lin *et al.* 2009; Li *et al.* 2012b 2013; Tang *et al.* 2015; Tan *et al.* 2015, 2018; Wang *et al.* 2015; Shang *et al.* 2015, 2016; Jamshed *et al.* 2016) to provide perspectives on the genetic control of various traits. While each contributes to our multi-dimensional understanding of the controls on phenotypes, (1) it is not immediately clear that interspecies QTL are useful in cotton breeding programs (Lin *et al.* 2009; Shang *et al.* 2015; Jamshed *et al.* 2016), and (2) inter-cultivar or inter-line crosses provide a limited perspective on the underlying genetic architecture leading to modern elite lines. The present QTL analysis was designed specifically to reveal the genetic architecture underlying the morphological transformation from wild to domesticated upland cotton, *G. hirsutum*. Like many of existing QTL analyses in cotton, our cross, while having allelic replication only in two environments, also demonstrates that the genomic differences that underlie many wild *vs.* cultivated characteristics are environmentally variable. Only about 18% of the QTL were shared across the two subpopulations. This variability is likely due to pleiotropic and environmentally labile regulatory factors and genetic interactions (Wittkopp *et al.* 2004; Coolon *et al.* 2014; Chen *et al.* 2015; Metzger *et al.* 2016; Rhoné *et al.* 2017; Signor and Nuzhdin 2018) playing a role in divergence between wild and domesticated species. This complexity is also increased by the allopolyploid nature of cotton, whose subgenomes evolved in isolation for 5-10 million years but now are reunited in a common nucleus, where they have coexisted for 1-2 million years. It is notable that, congruent with other QTL analyses, we find important fiber related QTL on the subgenome derived from the parent with the much shorter, inferior fiber (D genome). The involvement of the D-genome in the evolution of transgressive fiber phenotypes has been noted in multiple analyses, including for QTL (Jiang *et al.* 1998; Lacape *et al.* 2005; Han *et al.* 2006; Rong *et al.* 2007; Qin *et al.* 2008; Said *et al.* 2015b), expression (Hovav *et al.* 2008a; Yoo and Wendel 2014; Zhang *et al.* 2015; Fang *et al.* 2017b), and in selective genomic sweeps (Fang *et al.* 2017a, 2017c; Song *et al.* 2019), yet the underlying genetic basis for this phenomenon remains unclear. Further work using advanced populations in which individual QTL have been isolated in isogenic backgrounds, combined with a multi-omics or systems biology perspective, is one promising approach for developing a fuller understanding of cotton biology as well as the domestication process.
